# Rheumatic heart disease burden from 1990 to 2021: an updated analysis based on the global burden of disease study 2021

**DOI:** 10.3389/fpubh.2025.1674434

**Published:** 2025-12-10

**Authors:** Junjie Zhang, Ting Ren, Qiang Zhao

**Affiliations:** 1 Department of Cardiovascular Surgery, Ruijin Hospital Shanghai Jiao Tong University School of Medicine, Shanghai, China; 2 Department of Cardiothoracic Surgery, Wujin Hospital Affiliated with Jiangsu University, Changzhou, China; 3 Department of Cardiothoracic Surgery, Wujin Clinical College of Xuzhou Medical University, Changzhou, China

**Keywords:** rheumatic heart disease, global burden of disease, DALYs, mortality, incidence, prevalence, socio-demographic index, health inequality

## Abstract

**Background:**

Rheumatic heart disease (RHD) remains a significant contributor to cardiovascular morbidity and mortality, disproportionately affecting low- and middle-income countries. While global interventions have targeted the control of RHD, its burden and associated inequalities remain substantial. This study aims to evaluate temporal trends, socioeconomic disparities, and future projections of RHD burden across countries stratified by socio-demographic index (SDI).

**Methods:**

We analyzed data from the Global Burden of Disease Study spanning 1990 to 2021 to assess RHD incidence, prevalence, deaths, and disability-adjusted life years (DALYs). Inequality was quantified using the concentration index (CI) and slope index of inequality (SII). A decomposition analysis was conducted to attribute changes in RHD burden to population growth, population aging, and epidemiological change. Bayesian age-period-cohort models were used to forecast age-standardized rates of RHD burden through 2050.

**Results:**

From 1990 to 2021, the global age-standardized RHD burden declined substantially, particularly in high-SDI countries. However, concentration curves and inequality indices revealed persistent disparities, with low-SDI regions experiencing a disproportionately higher burden of disease. While inequalities in DALYs and mortality have improved modestly, the incidence and prevalence of the disease remain unequally distributed. Decomposition analysis showed that increases in absolute burden in low-SDI countries were primarily driven by population growth and aging, with minimal offset from epidemiological improvements. Future projections indicate continued declines in age-standardized death and DALYs rates, especially in high-SDI regions. In contrast, incidence and prevalence are expected to remain high in low-SDI countries.

**Conclusion:**

Despite global progress in reducing the overall burden of RHD, significant socioeconomic inequalities persist and are projected to continue. Strategies to reduce RHD must prioritize prevention, early intervention, and long-term care in low-resource settings.

## Introduction

Rheumatic heart disease (RHD) remains one of the most enduring global health challenges, particularly among socioeconomically disadvantaged populations (1). It is the long-term consequence of acute rheumatic fever following a Group A streptococcal infection, most commonly affecting children and young adults in low- and middle- income countries (2). As of 2019, there were over 40.5 million people worldwide living with RHD, with nearly 310,000 related deaths, resulting in approximately 10.67 million years of healthy life lost (3). Substantial progress has been made in reducing the overall burden of cardiovascular diseases, including RHD, through improved healthcare systems, the widespread use of antibiotics, and primary and secondary prevention strategies (4). However, these advancements have not been uniformly achieved across all regions. This study aims to quantify the past, current, and projected burden of RHD from 1990 to 2050 across Socio-demographic Index (SDI) quintiles, explore the underlying drivers of change, and evaluate global inequality trends using validated summary indices. Methodologically, this iteration advances prior GBD 2019 frameworks through the systematic integration of pandemic-era surveillance data (2020–2021) (5). This temporal extension enables a granular analysis of COVID-19’s collateral effects while stress-testing health system performance metrics during concurrent phases of chronic disease management and acute pandemic response. This study represents the first comprehensive investigation to leverage the GBD 2021 database for burden quantification, thereby filling a critical gap in cardiovascular epidemiology.

## Methods

### Data source

This study employs data from the GBD 2021 database, a methodological gold standard curated by the Institute for Health Metrics and Evaluation (IHME) at the University of Washington (available at https://ghdx.healthdata.org/gbd-2021). This multinational registry systematically aggregates epidemiological profiles of 369 diseases and injuries, alongside 87 modifiable risk factors, across 204 geopolitical entities (6). For this study, we extracted publicly available metrics related to RHD, including incidence, prevalence, mortality, and disability-adjusted life years (DALYs) (7). All estimates are reported as both absolute counts and age-standardized rates per 100,000 population, accompanied by corresponding 95% uncertainty intervals to ensure interpretability and statistical transparency.

### Estimation of incidence, prevalence, DALYs and mortality

RHD was identified according to the GBD cause list, categorized under Level 3, based on the International Classification of Diseases (ICD): ICD-10 codes I05–I09. To contextualize disease burden, the SDI—a composite indicator that integrates mean income per capita, total fertility rate, and average educational attainment at the national level—is used within the GBD framework (8). Countries are stratified along a five-tier SDI spectrum: high, high-middle, middle, middle-low, and low. Age-standardized rates are calculated relative to a globally defined reference population. Furthermore, projections of disease burden are accompanied by 95% uncertainty intervals, which reflect the probable range within which the actual parameter values are likely to lie. These intervals incorporate uncertainty arising from input data variability, model selection processes, and parameter estimation.

### Statistical methods

A longitudinal burden analysis (1990–2021) was conducted through demographic stratification (temporal, biological sex, and 5-year age cohorts) using Microsoft Excel 2019 (v16.0)—temporal trend visualization employed longitudinal trajectory mapping via epidemiological curve visualization protocols. Age-standardized rates were calculated by applying direct age standardization to the GBD 2021 reference population structure. The SDI served as a contextual framework for evaluating the relationship between levels of socio-demographic advancement and the burden of RHD. To identify the minimal attainable levels of prevalence, mortality, and DALYs, a frontier analysis was conducted. For forecasting, we applied Bayesian Age-Period-Cohort (BAPC) models using integrated nested Laplace approximations (9). These models project age-standardized rates of RHD burden from 2020 to 2050, incorporating uncertainty through posterior predictive distributions. Forecasts were stratified by SDI quintile to evaluate expected disparities under current trajectories.

## Results

### Prevalence of RHD

The global burden of RHD demonstrates significant inequalities and evolving epidemiological trends between 1990 and 2021. On a worldwide scale, age-standardized mortality rates (ASMR) declined substantially, with an annual reduction of −2.71% (95% UI: −2.75 to −2.67), while DALYs decreased by −2.52% annually (−2.55 to −2.48). Paradoxically, age-standardized prevalence (ASPR) and incidence (ASIR) rates both increased by 0.46% per year, indicating that despite improvements in survival, challenges in disease transmission and detection remain significant.

Marked sex-based disparities were evident, with females bearing a disproportionate burden across all metrics. Women exhibited 15.3% higher age-standardized DALYs rates (ASDR) (173.99 vs. 150.11 DALYs/100,000) and 22.9% higher ASPR (754.77 vs. 614.2 cases/100,000) compared to men, although the rates of mortality reduction were nearly identical between the sexes. Geographical and socio-demographic stratification highlighted profound inequities. Low-SDI regions had ASMR (9.54/100,000) and ASPR (1,184.23/100,000) that were 8.7 and 13.3 times higher, respectively, than those in high-SDI regions. While high-SDI regions experienced accelerated declines in ASIR (−0.93% per year), low-SDI regions saw a rising incidence (+0.27% per year), reflecting systemic failures in prevention efforts. South Asia emerged as the highest-burden region, with ASMR (14.88/100,000) 3.3-fold higher than the global average (4.47/100,000) and ASDR (453.58/100,000) 2.8-fold higher than the global average (162.12/100,000). Sub-Saharan Africa had the highest ASPR (1,404.41/100,000), underscoring severe healthcare access gaps (10, 11). The most rapid progress was observed in Central/Eastern Europe (ASMR: −4.8%/year) and Southeast/East Asia (ASDR: −4.35%/year). However, North Africa/Middle East faced rising ASIR (+0.21%/year), reflecting ongoing streptococcal transmission in vulnerable populations despite global mortality reductions (Table 1).

**Table 1:** Age-standardized rates and estimated annual percentage change for rheumatic heart disease burden (1990–2021) by SDI quintiles and super-regions.

Characteristic	ASMR (95% UI)		ASDR (95% UI)		ASPR (95% UI)		ASIR (95% UI)	
	2021	EAPC	2021	EAPC	2021	EAPC	2021	EAPC
Global	4.47(3.89, 5.31)	−2.71(−2.75, −2.67)	162.12(139.06, 190.52)	−2.52(−2.55, −2.48)	684.2(540.41, 848.9)	0.46 (0.42, 0.51)	50.74(40.10, 63.06)	0.46 (0.43, 0.5)
Male	4.22(3.54, 6.19)	−2.72(−2.79, −2.65)	150.11(125.15, 204.97)	−2.50(−2.56, −2.45)	614.2(482.58, 761.31)	0.47 (0.42, 0.52)	46.52(36.51, 57.84)	0.49 (0.45, 0.52)
Female	4.72(4.01, 5.82)	−2.72(−2.80, −2.65)	173.99 (148.21, 211.13)	−2.54(−2.62, −2.46)	754.77(600.82, 936.66)	0.47 (0.42, 0.52)	55.11(43.62, 68.68)	0.45 (0.41, 0.5)
SDI quintiles								
High SDI	1.1(0.93, 1.2)	−3.29(−3.57, −3.01)	24.68(22.15, 27.04)	−3.68(−4.02, −3.34)	89.18(81.38, 98.3)	−0.58(−0.71, −0.45)	6.78(6.18, 7.44)	−0.93(−1.1, −0.77)
High−middle SDI	2.11(1.77, 2.5)	−4.2(−4.38, −4.03)	61.45(52.63, 71.91)	−4.36(−4.5, −4.21)	323.97(270.17, 386.65)	−0.35(−0.44, −0.27)	20.36(16.96, 24.6)	−0.77(−0.83, −0.7)
Middle SDI	4.22(3.47, 5.19)	−3.83(−3.92, −3.74)	139.86(118.87, 165.24)	−3.45(−3.56, −3.34)	718.7(564.1, 889.1)	−0.01(−0.09, 0.06)	45.89(36.14, 57.57)	−0.1(−0.16, −0.03)
Low−middle SDI	10.68 (8.79, 14.85)	−1.98(−2.09, −1.88)	342.79(287.9, 445.93)	−2.16(−2.25, −2.08)	856.23(676.01, 1061.01)	0.25(0.24, 0.26)	60.46(47.74, 75.48)	0.24(0.23, 0.25)
Low SDI	9.54(7.29, 12.88)	−1.75(−1.96, −1.55)	311.11(250.77, 402.6)	−1.98(−2.12, −1.85)	1184.23(932.41, 1478.2)	0.27(0.24, 0.3)	84.89(66.11, 106.36)	0.27(0.25, 0.3)
Super-regions								
High−income	1.14(0.96, 1.25)	−2.74(−2.94, −2.53)	25.77(23.02, 28.6)	−2.97(−3.24, −2.71)	112.28(100.55, 127.01)	−0.29(−0.42, −0.17)	8.83(7.93, 9.89)	−0.46(−0.6, −0.32)
Central Europe, Eastern Europe, and Central Asia	1.52(1.4, 1.65)	−4.8(−4.98, −4.61)	58.81(51.92, 67.14)	−4.6(−4.78, −4.41)	324.4(275.14, 384.21)	0.02(−0.02, 0.06)	23.14(19.17, 27.81)	−0.16(−0.19, −0.12)
Latin America and Caribbean	0.87(0.76, 0.94)	−3.83(−3.94, −3.72)	71.49(54.99, 95.33)	−2.24(−2.33, −2.14)	901.82(717.65, 1115.87)	−0.04(−0.05, −0.02)	56.79(44.52, 71.23)	−0.08(−0.1, −0.06)
North Africa and Middle East	1.9(1.62, 2.23)	−3.11(−3.21, −3)	86.78(71.3, 104.16)	−2.98(−3.06, −2.9)	540.77(428.63, 660.44)	0.18(0.14, 0.22)	37.29(29.57, 46.07)	0.21(0.17, 0.25)
South Asia	14.88(12.23, 20.01)	−2.01(−2.13, −1.88)	453.58(380.1, 580.89)	−2.19(−2.3, −2.07)	732.2(575.83, 916.47)	0.2(0.17, 0.24)	51.33(40.39, 64.3)	0.16(0.12, 0.2)
Southeast Asia, East Asia, and Oceania	3.61(2.94, 4.48)	−4.63(−4.75, −4.51)	106.19(89.37, 125.79)	−4.35(−4.47, −4.23)	546.86(435.47, 670.83)	−0.24(−0.36, −0.12)	35.47(28.25, 43.86)	−0.45(−0.56, −0.33)
Sub−Saharan Africa	2.85(2.47, 3.46)	−2.92(−2.96, −2.87)	145.3(114.29, 186.8)	−2.03(−2.08, −1.98)	1404.41(1109.35, 1758.62)	0.15(0.13, 0.16)	96.14(74.77, 120.97)	0.12(0.11, 0.14)

EAPC, estimated annual percentage change 1990–2021; ASMR, age-standardized mortality rate; ASDR, age-standardized disability-adjusted life years rate; ASPR, age-standardized prevalence rate; ASIR, age-standardized incidence rate; UI, uncertainty interval; SDI, socio-demographic index.

The regional analysis of RHD burden reveals profound disparities in epidemiological trends and disease impact across 21 subregions (Supplementary Table 1). While global mortality and DALYs showed significant declines, persistently high-burden regions highlight unresolved healthcare inequities. South Asia emerged as the highest-burden region, with 2021 ASMR of 14.88 deaths per 100,000 (representing 3.3 times the global average of 4.47) and ASDR of 453.58 per 100,000 (2.8 times the global average of 162.12). In contrast, high-income regions reported the lowest burden, with high-income North America recording an ASMR of 0.85 and ASDR of 22.72. Sub-Saharan Africa faced severe prevalence challenges, particularly in Central Sub-Saharan Africa (ASPR: 1,665.95 per 100,000) and Eastern Sub-Saharan Africa (ASPR: 1,584.28 per 100,000), reflecting fragile health systems and limited access to penicillin prophylaxis (12).

Regions with robust primary care integration experienced significant reductions in mortality. Central Latin America achieved the fastest decline in ASMR (Estimated Annual Percentage Change (EAPC): −6.06%/year), followed by Eastern Europe (−5.95%/year) and Central Europe (−5.33%/year). These successes contrast with Oceania, where ASMR remained critically high at 11.71 per 100,000, despite modest declines (−1.61%/year), underscoring the urgent need for tailored interventions in island nations. The persistently high ASMR in Oceania reflects the synergistic effects of healthcare access barriers and housing-related social determinants. Severe household overcrowding (more than eight persons per bedroom) significantly increases the incidence of acute rheumatic fever. These factors, compounded by geographic isolation, leave many high-burden communities without adequate clinical services. Addressing this challenge requires integrated interventions that combine housing improvements with mobile healthcare delivery (13–15).

Incidence and prevalence trends revealed divergent patterns. Southeast Asia, East Asia, and Oceania collectively reduced ASIR (−0.45%/year) through improved diagnosis and treatment, while the Caribbean saw rising ASIR (+0.26%/year) and ASPR (+0.21%/year), signaling ongoing transmission risks. Regional sex disparities were evident in all regions, with females experiencing 15–23% higher prevalence and mortality than males, highlighting the need for gender-sensitive prevention strategies.

Geographical heterogeneity in progress was stark. Tropical Latin America stabilized the ASPR (EAPC: 0%/year) through enhanced secondary prevention efforts, whereas North Africa and the Middle East faced a rising incidence (ASIR +0.21%/year), exacerbated by population growth and barriers to antibiotic access (16). South Asia’s moderate ASMR reduction (−2.01%/year) lagged behind its economic development, revealing gaps in rheumatic fever control. In contrast, Central Europe’s rapid decline in ASIR (−2.31%/year) underscored the benefits of centralized registries and widespread availability of penicillin (17–19). These regional patterns, underscore that, despite global improvements in mortality, RHD remains entrenched in low-resource settings, requiring region-specific escalation of primary prevention, equitable access to benzathine penicillin, and substantial investment in healthcare systems to address the triple burden of poverty, limited diagnostics, and delayed care (Supplementary Tables 1, 2).

### Age-sex burden and socioeconomic inequities in RHD

**Figure 1:**
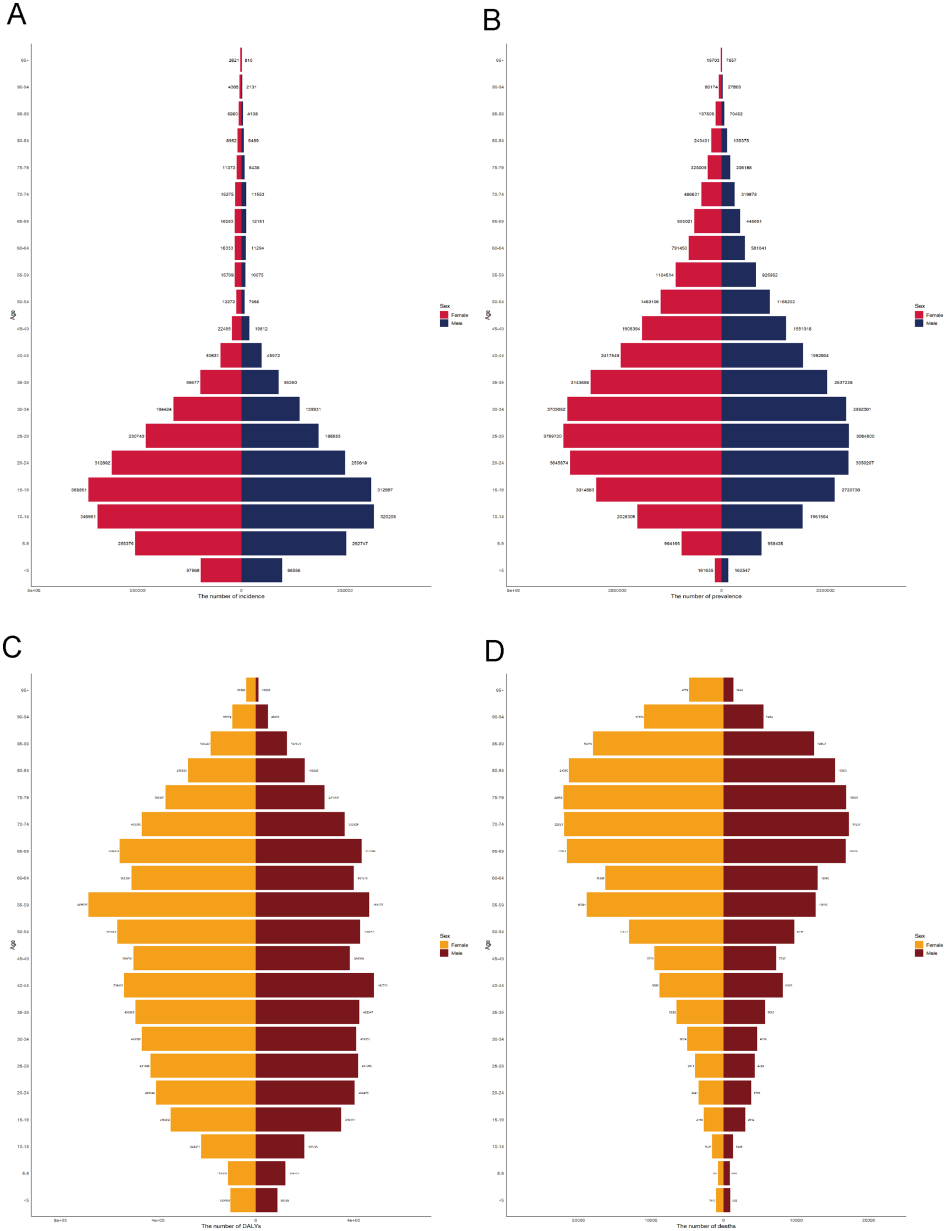
Age and sex distribution of rheumatic heart disease burden globally in 2021. **(A)** Incident cases, **(B)** Prevalent cases, **(C)** DALYs, **(D)** Deaths.

In 2021, the burden of RHD showed significant heterogeneity across age and sex groups. The absolute number of DALYs peaked in middle-aged and older adults, with females experiencing a higher burden than males, particularly in the 55–69 age range. Mortality followed a similar trend, increasing with age and disproportionately affecting females. Incident cases were most concentrated among adolescents and young adults (ages 10–29), with a consistently higher incidence observed in females across all age groups. The prevalence was highest among individuals aged 20–50 years, with females bearing a greater burden of disease. These findings underscore persistent gender disparities and age-specific patterns of RHD transmission and progression (Figure 1).

**Figure 2:**
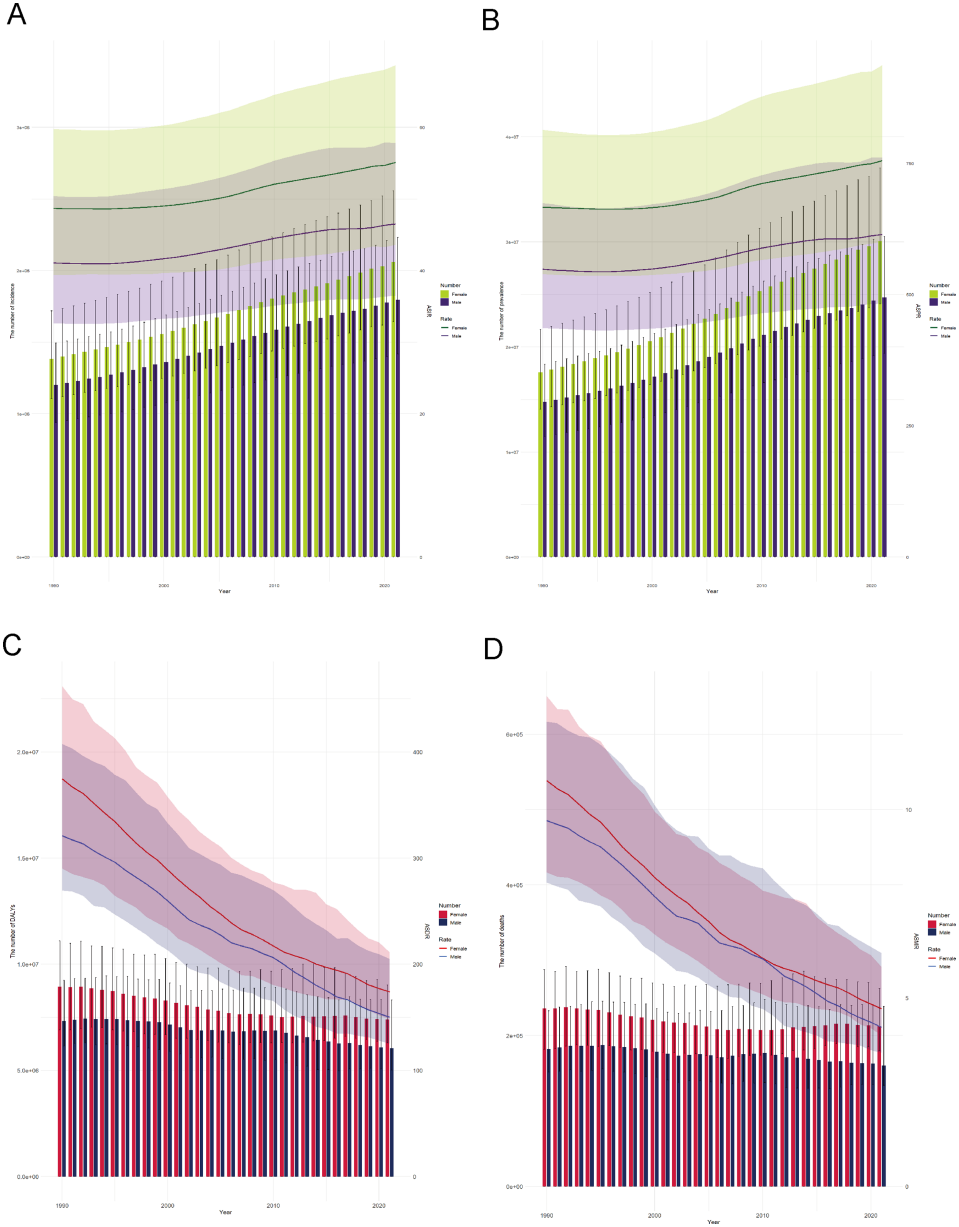
Global trends in rheumatic heart disease burden from 1990 to 2021 by sex. **(A)** Incidence, **(B)** Prevalence, **(C)** DALYs, **(D)** Deaths.

Between 1990 and 2021, the global burden of RHD exhibited divergent trends in mortality and morbidity. Both DALYs and deaths declined significantly over time, with age-standardized rates showing a marked reduction in both sexes, although the absolute burden remained higher in females. In contrast, the global number of incident and prevalent RHD cases steadily increased. While ASIR remained relatively stable, ASPR gradually rose—most notably among females—indicating both improved survival and ongoing disease transmission. These temporal trends illustrate a shifting RHD landscape, characterized by a decline in fatality but an expansion in disease persistence, necessitating a renewed emphasis on primary prevention, early detection, and equitable access to care (Figure 2, Supplementary Figure 1).

### Socioeconomic disparities in RHD between high and low income regions

**Figure 3:**
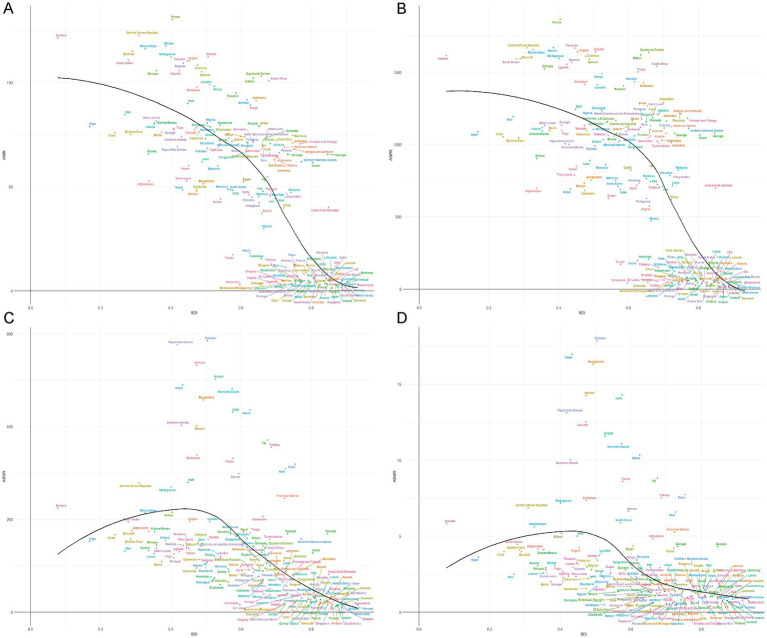
National distribution of rheumatic heart disease burden in 2021 by Socio-Demographic Index. **(A)** Age-standardized incidence rate (ASIR), **(B)** Age-standardized prevalence rate (ASPR), **(C)** Age-standardized DALYs rate (ASDR), **(D)** Age-standardized mortality rate (ASMR).

Countries with lower SDI experienced significantly higher age-standardized rates of DALYs, incidence, mortality, and prevalence. High-income countries exhibited lower rates of these outcomes, demonstrating the protective role of higher development levels and better access to healthcare. However, even within middle-income regions, RHD remains a significant public health issue, with countries in Southeast Asia and Sub-Saharan Africa showing elevated prevalence and incidence rates despite improvements in survival (Figure 3).

**Figure 4:**
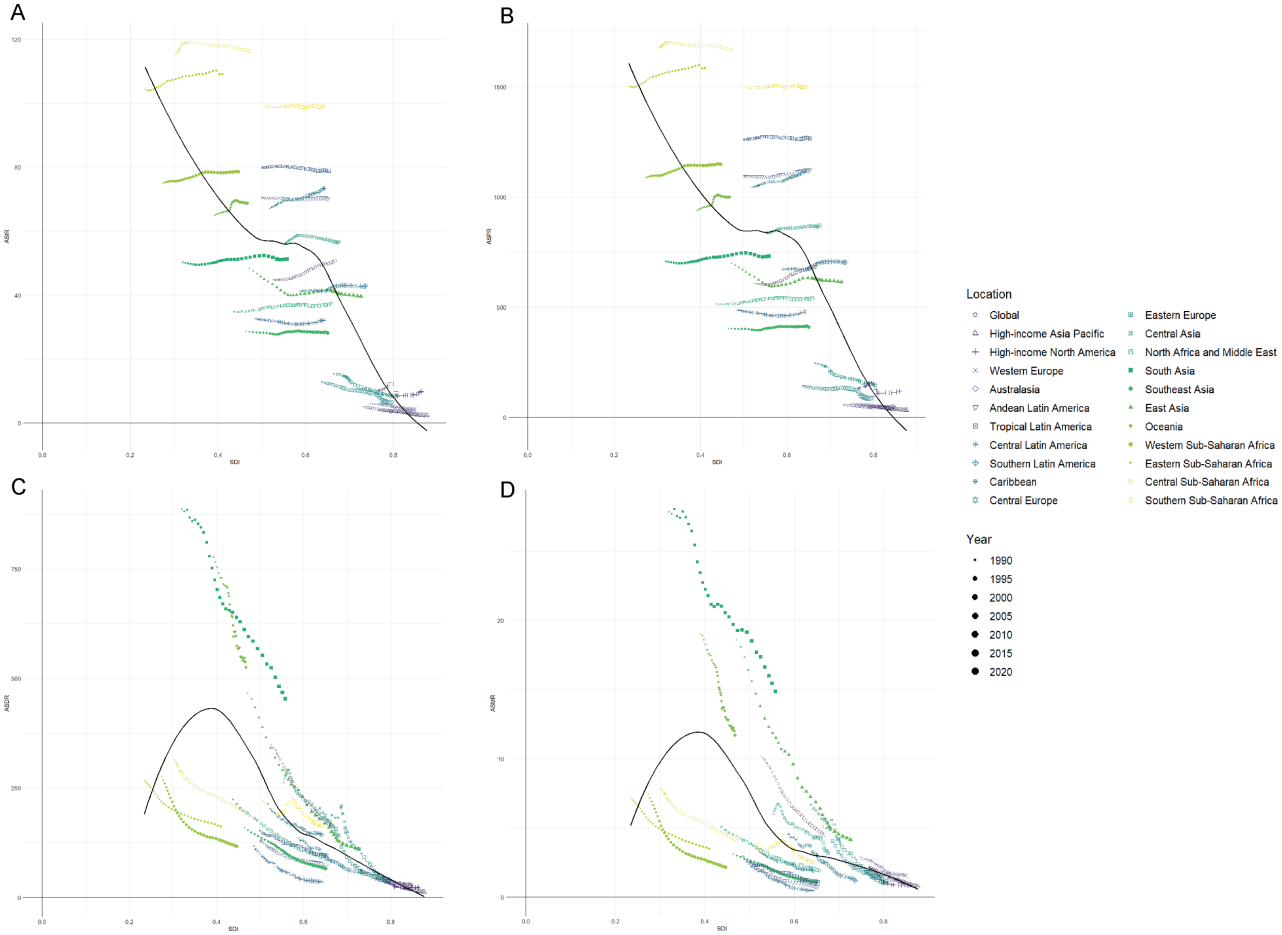
Age-standardized burden of rheumatic heart disease by region and Socio-demographic Index (SDI). 1990–2020. **(A)** Age-standardized incidence rate (ASIR), **(B)** Age-standardized prevalence rate (ASPR), **(C)** Age-standardized DALYs rate (ASDR), **(D)** Age-standardized mortality rate (ASMR).

Over the past three decades, high-SDI regions experienced noticeable declines in RHD burden across all metrics, driven by advancements in healthcare and disease management. In contrast, countries with lower SDI, particularly in South Asia and Sub-Saharan Africa, showed slower reductions in DALYs and mortality rates, with some regions stagnating or improving at a slower pace. The prevalence of RHD remained high in many lower-income areas, indicating that, while mortality has decreased due to better survival, the incidence and long-term burden of the disease continue to affect these regions disproportionately. These patterns underscore the ongoing challenges in reducing the RHD burden, particularly in areas with limited healthcare resources and inadequate access to preventive care (Figure 4).

Over this period, significant reductions in age-standardized rates of DALYs, incidence, mortality, and prevalence have been observed in high-income regions such as North America and Europe. These regions have demonstrated significant improvements in healthcare access, disease management, and prevention, resulting in a steady decline in the RHD burden. In contrast, low- and middle-income regions, particularly Sub-Saharan Africa and South Asia, continue to experience a higher burden of RHD, with only gradual improvements over time. Despite some decline in mortality rates, these regions still struggle with high rates of incidence, prevalence, and DALYs (Supplementary Figure 2).

High-income regions consistently show the lowest RHD burden across all indicators. In contrast, Sub-Saharan Africa and South Asia maintain higher levels of disease burden, particularly in terms of mortality and DALYs. Southeast Asia, East Asia, and Latin America have seen mixed improvements, with some reductions in incidence and prevalence, but still maintain higher-than-average rates compared to high-income regions. The data underscore global inequities in RHD burden and highlight the need for targeted interventions in areas where healthcare access and disease prevention remain significant challenges. These findings stress the importance of addressing persistent disparities in disease management and access to care, particularly in low- and middle-income countries (Supplementary Figure 3).

### Global distribution and temporal trends in RHD burden

**Figure 5:**
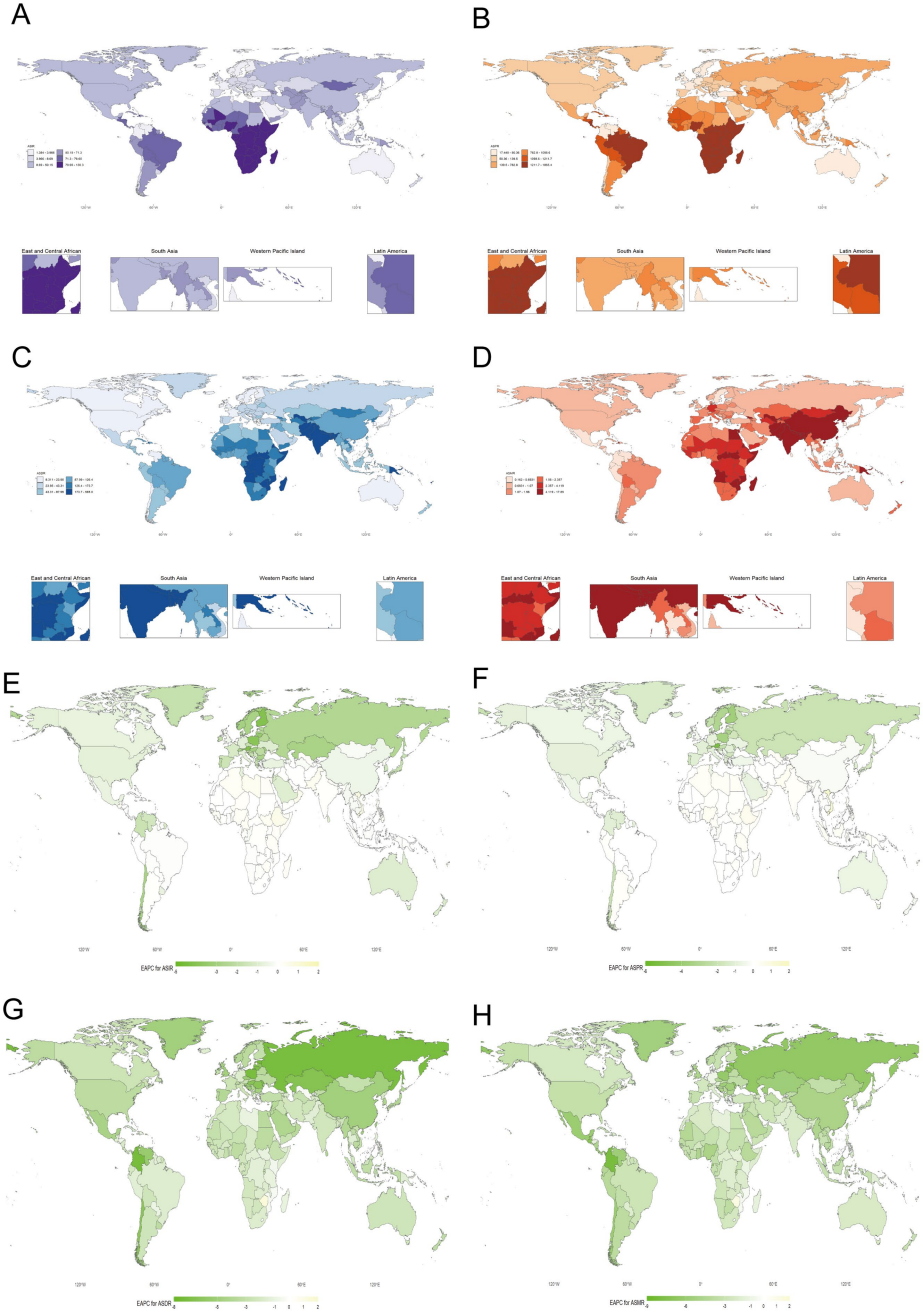
Global distribution of RHD Burden (2021) and temporal trends (1990–2021). **(A)** Age-standardized incidence rate (ASIR) (2021), **(B)** Age-standardized prevalence rate (ASPR) (2021), **(C)** Age-standardized DALYs rate (ASDR) (2021), **(D)** Age-standardized mortality rate (ASMR) (2021), **(E)** EAPC for ASIR (1990–2021), **(F)** EAPC for ASPR (1990–2021), **(G)** EAPC for ASDR (1990–2021), **(H)** EAPC for ASMR (1990–2021).

The global distribution in 2021 reveals that regions such as Sub-Saharan Africa, South Asia, and parts of Southeast Asia bear the highest burden of RHD, exhibiting elevated values across ASDR, ASIR, ASMR, and ASPR. In contrast, high-income regions in Europe and North America report significantly lower rates across all indicators, reflecting better access to healthcare, disease prevention, and management. The EAPCs from 1990 to 2021 demonstrate substantial improvements in high-income countries, where rates of DALYs, incidence, mortality, and prevalence have declined significantly. However, low- and middle-income regions, particularly those in South Asia and Sub-Saharan Africa, continue to experience slow reductions or stagnation in the burden of RHD. Some areas have even observed an increase in incidence rates (Figure 5).

### Socioeconomic inequality in RHD burden across countries and time

**Figure 6:**
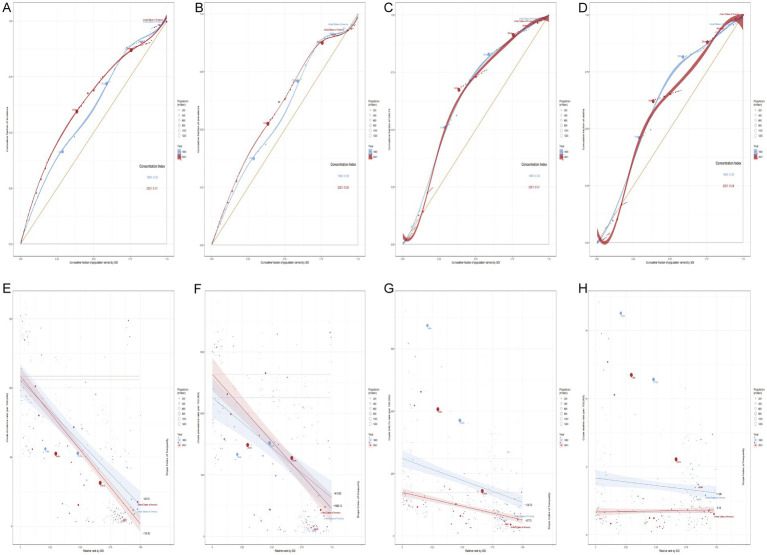
Inequality in Rheumatic Heart Disease Burden by SDI Using CI and SII Metrics (1990–2021). **(A–D)** Concentration Index (CI) of RHD burden in 2021: **(A)** Incidence, **(B)** Prevalence, **(C)** Disability-Adjusted Life Years (DALYs), **(D)** Deaths; **(E–H)** Slope Index of Inequality (SII) of RHD burden in 2021: **(E)** Incidence, **(F)** Prevalence, **(G)** DALYs, **(H)** Deaths.

The burden is heavily concentrated in low-SDI countries in both 1990 and 2021, with only a modest shift toward greater equality over time. The CI has slightly declined, indicating some improvement; however, the overall curve remains far from the line of equality. This pattern underscores that RHD disproportionately affects the world’s poorest populations, and this disparity persists despite global efforts to reduce the absolute burden. Crude DALYs and incidence rates remain strongly and negatively correlated with SDI, signaling persistent inequality, though the gradients have slightly flattened over time. Mortality inequality has narrowed significantly, with the SII shifting from negative to nearly neutral, indicating that death rates due to RHD are becoming less stratified by socioeconomic status. Conversely, prevalence continues to exhibit steep inequality, with lower-SDI countries experiencing increasingly disproportionate caseloads. The trend for prevalence reflects a concerning worsening trajectory, as the inequality gap continues to widen. This divergence suggests that, while some dimensions of the RHD burden—such as mortality—have become more equitable, others, particularly long-term disease prevalence, exhibit widening disparities. This is likely due to insufficient access to early intervention and sustained healthcare in regions with a low SDI. Despite meaningful global health interventions aimed at reducing certain aspects of RHD inequality, much of the burden still disproportionately affects the world’s most disadvantaged populations. The findings underscore the critical need for tailored policies that not only focus on mortality but also prioritize prevention, early diagnosis, and long-term disease management in low- and middle-income countries (Figure 6 and Supplementary Figure 4).

### Decomposition of absolute changes in RHD burden across SDI quintiles

Across all metrics, the most substantial increases in burden occurred in low- and low-middle-SDI countries, with demographic factors serving as the primary contributors. Population growth consistently emerged as the dominant driver, particularly in lower-SDI regions, where rapidly expanding populations led to significant increases in the absolute number of DALYs, deaths, and new or existing RHD cases. Population aging also played an important role, particularly in the middle SDI quintile, where changes in age structure intensified the burden, despite some improvements in disease management.

The impact of epidemiological change—reflecting health interventions, risk factor reduction, and care quality—was more mixed. In high- and high-middle- SDI countries, epidemiological improvements contributed to a decrease in the burden, effectively counteracting the pressures of aging and population growth. Conversely, in lower-SDI settings, the effect of epidemiological change was either marginal or negative, indicating limited progress in the prevention and control of RHD.

**Figure 7:**
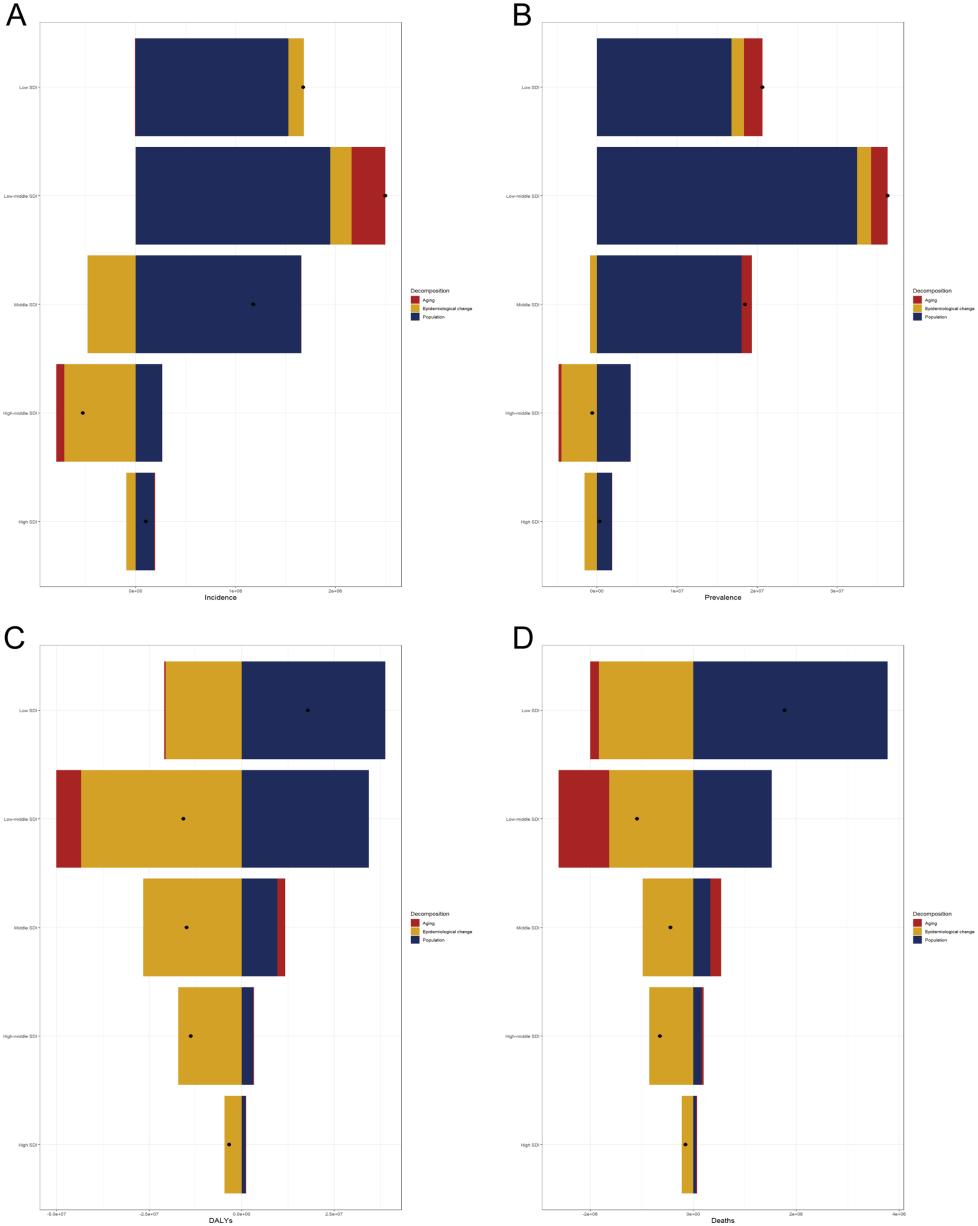
Rheumatic heart disease burden between 1990 and 2021 by SDI quintile and contributing factors. **(A)** Incidence, **(B)** Prevalence, **(C)** DALYs, **(D)** Deaths.

Overall, this analysis highlights that while higher-SDI countries have primarily managed to curb or stabilize their RHD burden through epidemiological gains, the burden in low-SDI countries continues to rise, primarily due to demographic expansion and insufficient health system advancements. Addressing these disparities will require strategic investment in prevention, early treatment, and health infrastructure in the most affected regions (Figure 7).

### Forecasted trends in RHD burden through 2050

ASDR is projected to continue its long-term global decline, with the most substantial reductions expected in high-SDI countries. This trend reflects ongoing success in prevention, treatment, and access to healthcare. In contrast, low-SDI countries are projected to experience more modest declines, indicating persistent gaps in health system capacity and coverage of interventions. Regarding ASIR, the global trend appears relatively flat, with a slight decrease in high-SDI regions but possible stagnation or even an increase in lower-SDI countries. These projections suggest that without intensified prevention strategies, new RHD cases may continue to rise at high rates, particularly in vulnerable populations.

The forecasted ASMR follows a downward trajectory, particularly in middle- and high-SDI regions. However, the pace of decline is slower in low-SDI countries, reflecting continued inequities in timely diagnosis and effective care. While global mortality is expected to improve, the unequal rate of progress highlights the need for targeted interventions in resource-poor settings.

**Figure 8:**
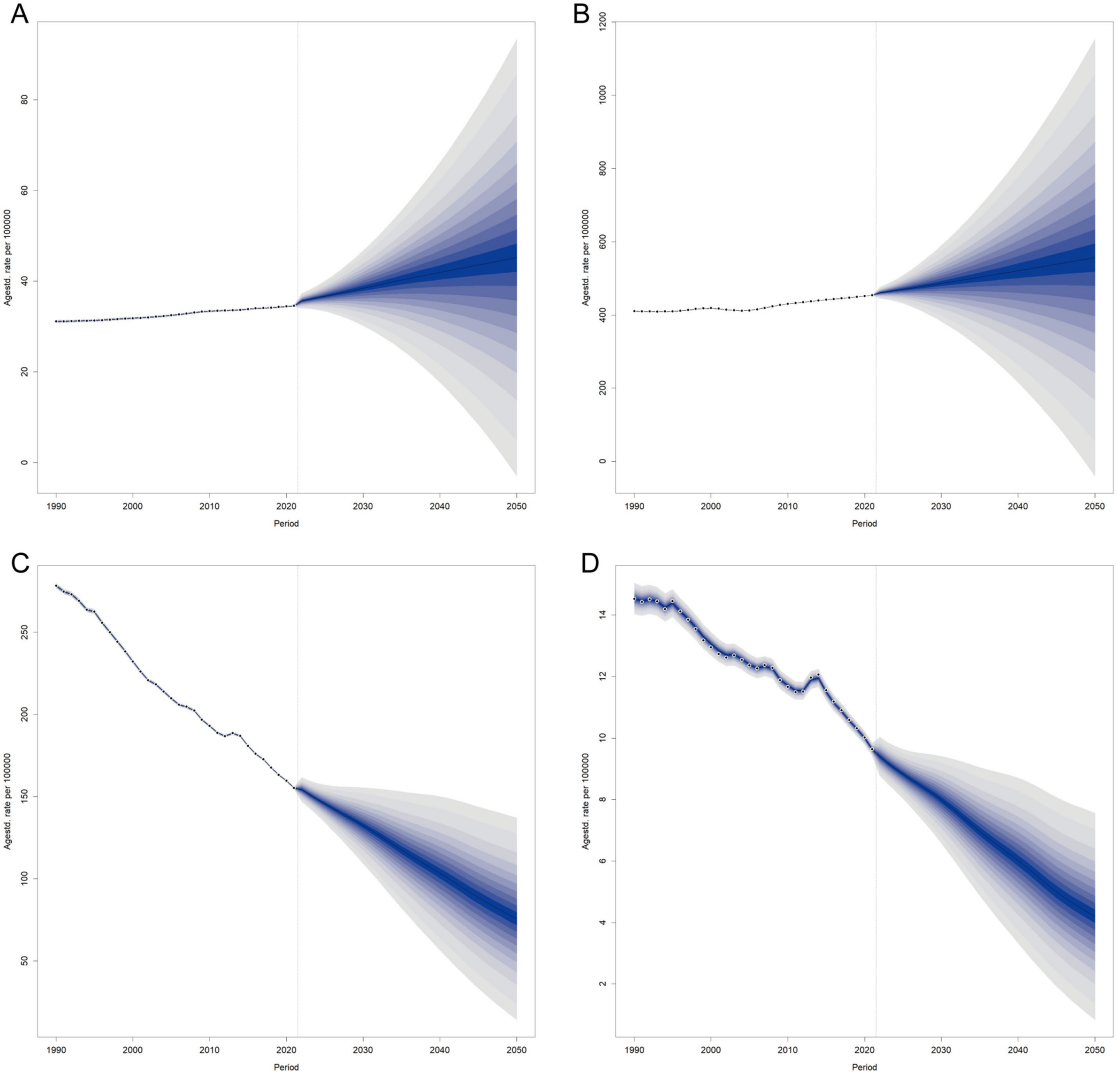
Projected age-standardized rates for RHD metrics by SDI quintile from 1990 to 2050. **(A)** Age-standardized incidence rate (ASIR), **(B)** Age-standardized prevalence rate (ASPR), **(C)** Age-standardized DALYs rate (ASDR), **(D)** Age-standardized mortality rate (ASMR).

ASPR is projected to rise slightly or remain stable in low-SDI regions, driven by improved survival rates without a corresponding reduction in incidence. In contrast, high-SDI countries may exhibit stabilization or a modest decrease in prevalence. This suggests a shift in the burden toward chronic disease management, particularly in settings lacking comprehensive long-term care systems (Figure 8).

## Discussion

This study presents a systematic assessment of the global burden of RHD from 1990 to 2021, with a stratified analysis based on the SDI and projections up to 2050. While the global RHD burden has declined overall, significant disparities remain between high-SDI and low-SDI countries. High-SDI countries have made notable improvements in ASMR, ASPR, and DALYs. In contrast, middle- and low-SDI countries have seen only modest progress. The primary drivers of the increasing RHD burden in low-SDI regions are population growth and aging, with limited epidemiological improvements that have failed to reduce the disease’s progression meaningfully. Without targeted interventions, these disparities are expected to widen, with low-SDI countries continuing to bear the highest global disease burden.

Analysis of the contributing factors reveals that population growth and aging have played a decisive role in the increased RHD burden in low-income regions. These regions’ modest advances in ASPR, ASMR, and ASDR have been offset by shifts in demographic structure. In contrast, wealthier areas have mitigated the negative impacts of population growth through effective epidemiological measures, such as early screening and treatment strategies. Additionally, a significant inverse relationship is observed between RHD indicators and SDI: higher SDI corresponds to lower ASPR, ASMR, and ASDR. In low-SDI countries, RHD predominantly affects younger populations, reflecting a demographic structure characterized by higher fertility rates and shorter life expectancy.

In high-SDI countries, RHD patients tend to be older due to longer life expectancy. This trend, driven by population aging, has been mitigated by the stronger healthcare infrastructure and medical resources in these countries, which enable the implementation of evidence-based interventions that slow the rise in the RHD burden (20). However, these improvements are still constrained by population growth and aging. Thus, the study emphasizes the importance for low-SDI countries to prioritize reproductive health policies and enhance primary healthcare infrastructure to improve life expectancy and disease control capabilities.

Gender differences are another critical aspect of this study. Previous research has consistently shown that RHD disproportionately affects women, particularly those of childbearing age (21). This study further confirms this and demonstrates that young women in low-SDI countries bear a higher RHD burden, likely due to factors such as limited maternal healthcare resources and the absence of early screening programs. In high-SDI countries, outcomes for women have notably improved, supported by more advanced epidemiological transitions and better population structure management (22). Given the differences in fertility rates and maternal health, it is recommended that all countries enhance routine echocardiographic screening and preventive measures for women, especially those of childbearing age.

Finally, this study highlights that in low-SDI countries, RHD predominantly affects children and adolescents, as evidenced by a higher ASPR, while older populations show higher ASMR and ASDR. This pattern is closely linked to factors such as poverty, limited educational opportunities, low health literacy, and inadequate healthcare resources. These barriers are especially severe in remote and underserved areas. Therefore, global health initiatives should prioritize early intervention and accessible healthcare services for children and adolescents, expand screening programs, improve diagnostic timeliness, and ensure continuous treatment to reduce RHD-related morbidity and mortality (4).

Our findings align with previous research on the persistent global inequality in the burden of RHD but extend the understanding by incorporating recent data and projections (3). Similar to earlier studies, our results confirm that RHD remains predominantly concentrated in low- and middle-income countries, where both incidence and prevalence rates remain high, despite global efforts in disease prevention and control (23). Furthermore, the role of demographic factors population growth and aging, primarily—is a driver of increased RHD burden, suggesting that while healthcare improvements are critical, demographic changes must also be addressed to combat the growing burden of RHD in resource-poor settings effectively. Our decomposition analysis reinforces these findings by demonstrating that while population growth and aging significantly contribute to the RHD burden, the lack of substantial epidemiological improvements in lower-SDI countries continues to hinder progress.

While this study provides valuable insights into the global burden of RHD, it is not without limitations. First, the reliance on existing data from the GBD study means that the quality and completeness of the estimates are subject to the inherent limitations of the GBD methodology, which depends on the availability of health data and model assumptions (24). Second, despite the use of Bayesian age-period-cohort models to project future trends, the inherent uncertainty in forecasting long-term outcomes remains a challenge, as numerous unpredictable factors, such as changes in healthcare access, emerging risk factors, and shifts in global health policy, influence projections (25). Furthermore, the decomposition analysis, although helpful in understanding the drivers of burden, does not account for potential confounders, such as the impact of climate change, geopolitical instability, or specific interventions that may influence RHD burden in the future (26). Finally, this study primarily focuses on national-level data, which may mask critical within-country variations in RHD burden, particularly in large, heterogeneous countries with significant regional disparities.

## Conclusion

RHD remains a critical global health challenge, with substantial disparities in disease burden between high- and low-SDI countries. Despite international efforts to reduce RHD through prevention and treatment, significant inequalities persist, particularly in low- and middle-income regions, where the burden of disease continues to grow. Ultimately, addressing RHD will require coordinated global efforts, enhanced healthcare infrastructure, and tailored interventions that are responsive to the unique needs of low-resource settings.

## Data Availability

The original contributions presented in the study are included in the article/Supplementary material, further inquiries can be directed to the corresponding authors.

## Supplementary material

SUPPLEMENTARY FIGURE 1 Age distribution of rheumatic heart disease burden globally in 2021 **(A)** Incident cases, **(B)** Prevalent cases, **(C)** DALYs, **(D)** Deaths.

SUPPLEMENTARY FIGURE 2 Temporal trends of age-standardized RHD burden by SDI categories. **(A)** Age-standardized incidence rate (ASIR), **(B)** Age-standardized prevalence rate (ASPR), **(C)** Age-standardized DALYs rate (ASDR), **(D)** Age-standardized mortality rate (ASMR).

SUPPLEMENTARY FIGURE 3 Age-standardized RHD burden by region and SDI Level (1990-2020). **(A)** Age-standardized incidence rate (ASIR), **(B)** Age-standardized prevalence rate (ASPR), **(C)** Age-standardized DALYs rate (ASDR), **(D)** Age-standardized mortality rate (ASMR).

SUPPLEMENTARY FIGURE 4 Temporal trends of index of inequality (SII) from 1990 to 2021. **(A)** Incidence, **(B)** Prevalence, **(C)** DALYs, **(D)** Deaths.
