# Supplementary figures and images for: Rheumatic heart disease burden from 1990 to 2021: an updated analysis based on the global burden of disease study 2021
Source: Front Public Health. 2025 Dec 10;13:1674434. doi: 10.3389/fpubh.2025.1674434 (PMC12727567; doi:10.3389/fpubh.2025.1674434)

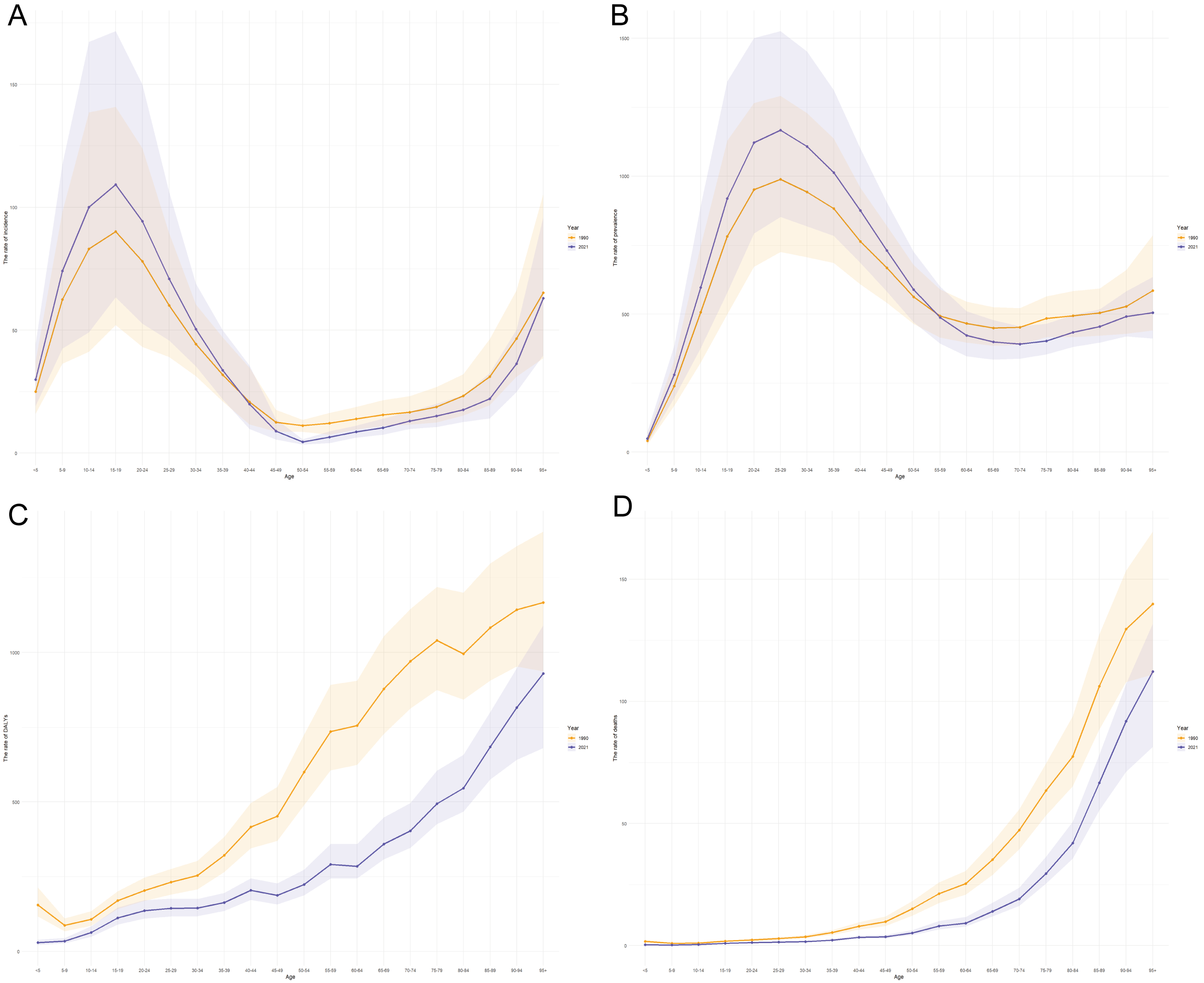

Supplement: SUPPLEMENTARY FIGURE 1 — Age distribution of rheumatic heart disease burden globally in 2021 (A) Incident cases, (B) Prevalent cases, (C) DALYs, (D) Deaths. [file Image_1.tif]

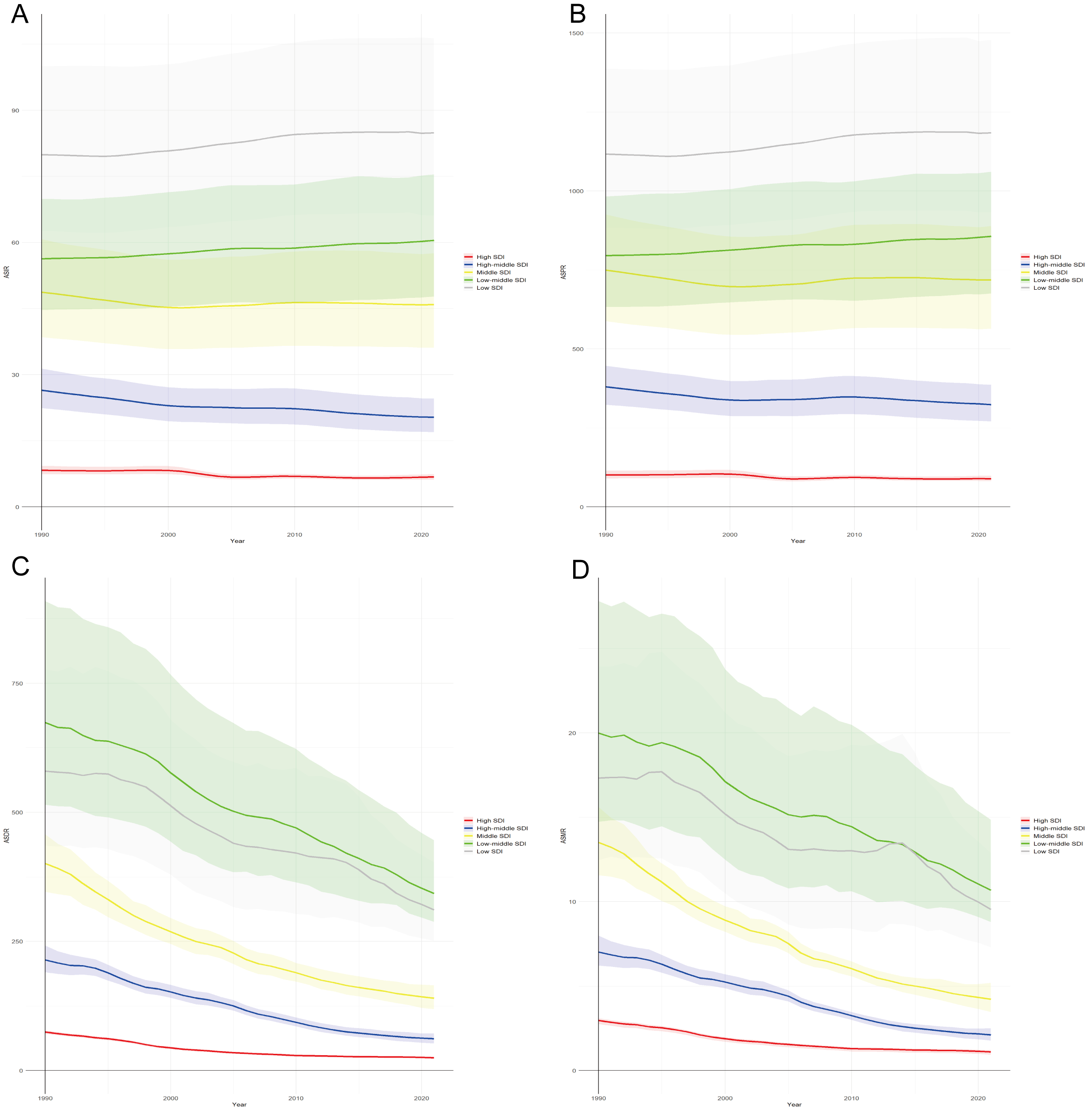

Supplement: SUPPLEMENTARY FIGURE 2 — Temporal trends of age-standardized RHD burden by SDI categories. (A) Age-standardized incidence rate (ASIR), (B) Age-standardized prevalence rate (ASPR), (C) Age-standardized DALYs rate (ASDR), (D) Age-standardized mortality rate (ASMR). [file Image_2.tif]

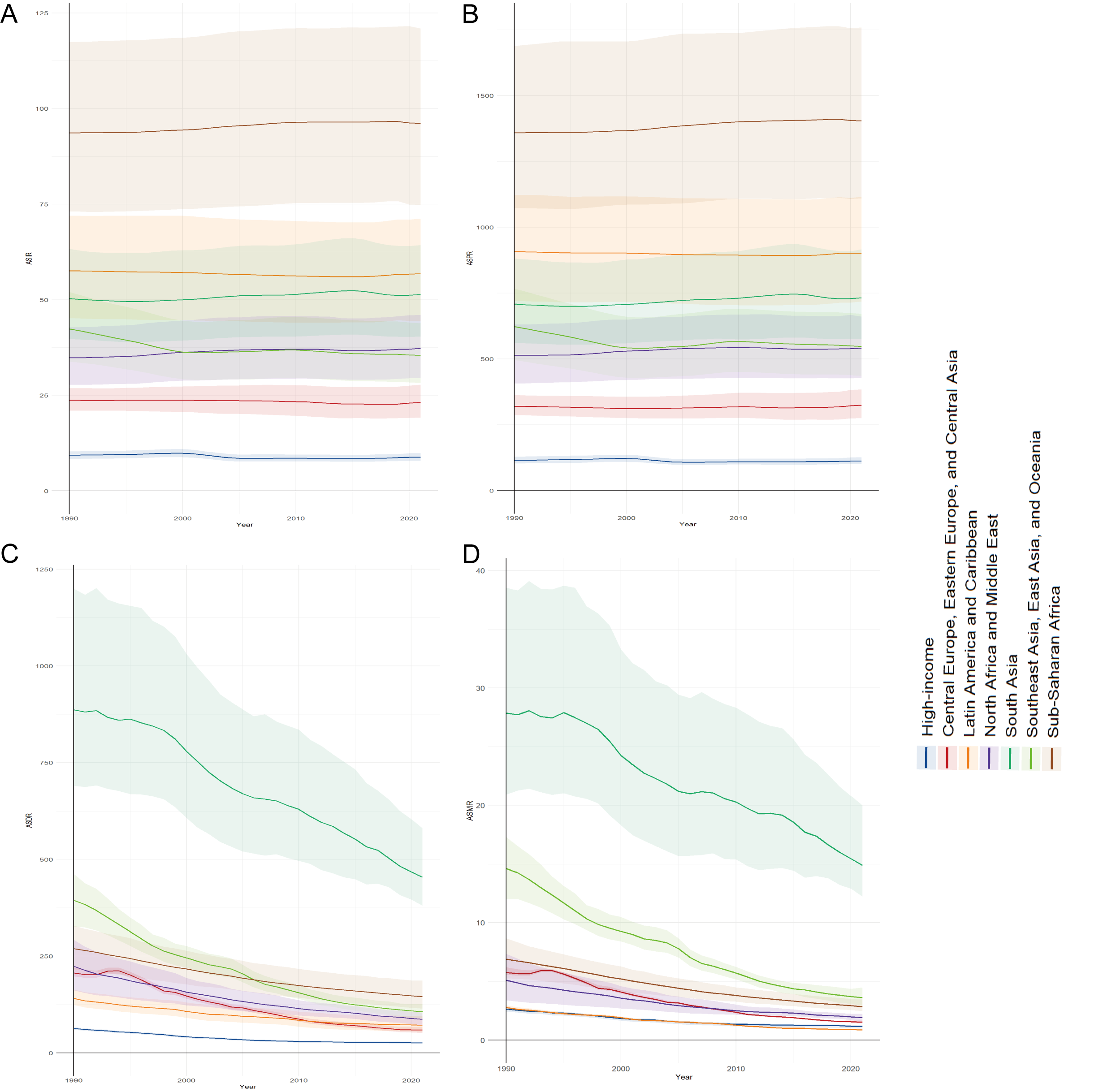

Supplement: SUPPLEMENTARY FIGURE 3 — Age-standardized RHD burden by region and SDI Level (1990-2020). (A) Age-standardized incidence rate (ASIR), (B) Age-standardized prevalence rate (ASPR), (C) Age-standardized DALYs rate (ASDR), (D) Age-standardized mortality rate (ASMR). [file Image_3.tif]

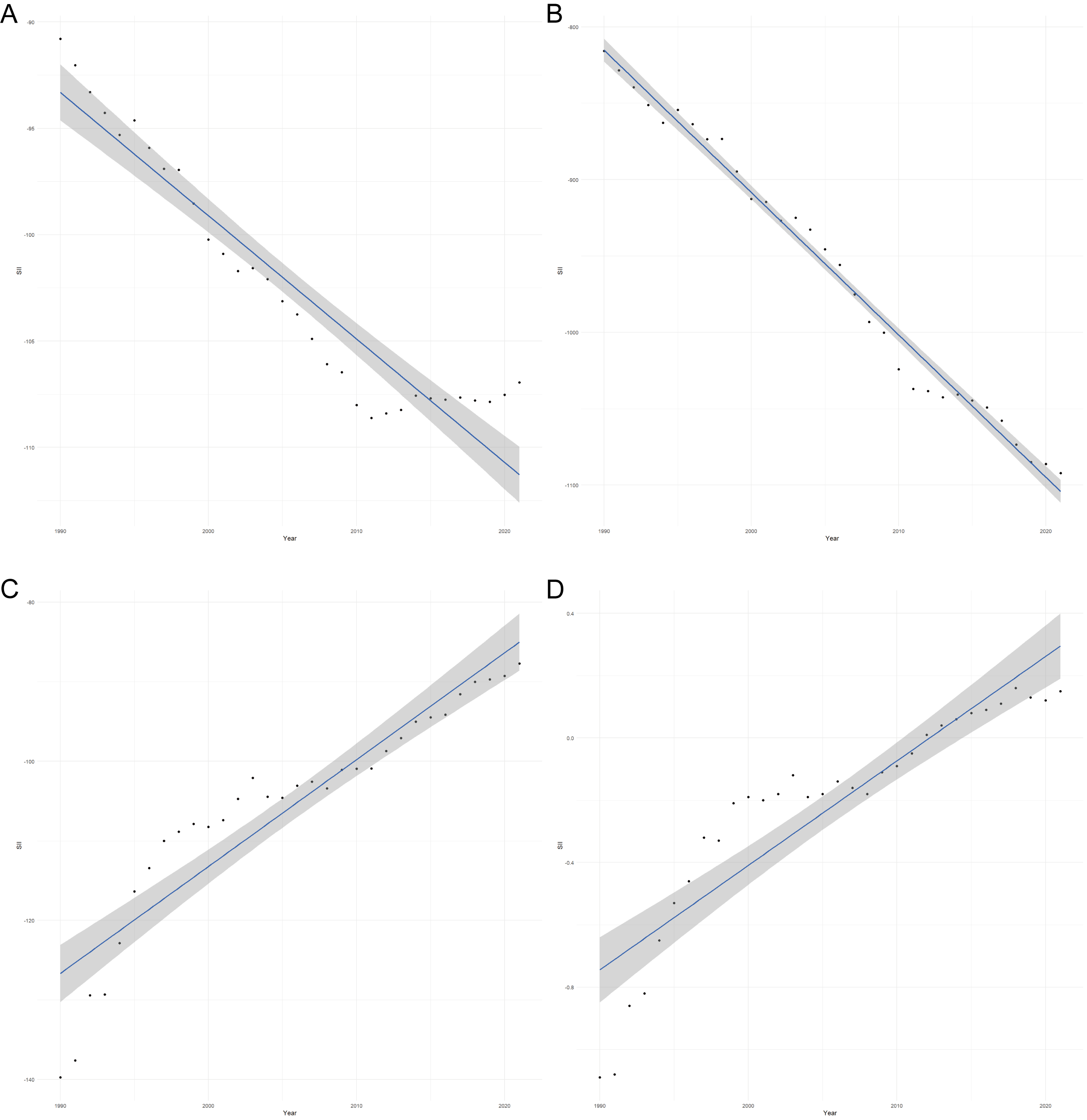

Supplement: SUPPLEMENTARY FIGURE 4 — Temporal trends of index of inequality (SII) from 1990 to 2021. (A) Incidence, (B) Prevalence, (C) DALYs, (D) Deaths. [file Image_4.tif]
