# Supplementary material for: Rheumatic heart disease burden from 1990 to 2021: an updated analysis based on the global burden of disease study 2021
Source: Front Public Health. 2025 Dec 10;13:1674434. doi: 10.3389/fpubh.2025.1674434 (PMC12727567; doi:10.3389/fpubh.2025.1674434)
Supplement: Supplementary file 5 [file Table_1.docx]

**Supplemental Table 1.** **Age-standardised rates and estimated annual percentage change for rheumatic heart disease burden (1990–2021) across 21 subregions**

|  | **ASMR (95% UI)** | |  | **ASDR (95% UI)** | |  | **ASPR (95% UI)** | |  | **ASIR (95% UI)** | |
| --- | --- | --- | --- | --- | --- | --- | --- | --- | --- | --- | --- |
|  | **2021** | **EAPC** |  | **2021** | **EAPC** |  | **2021** | **EAPC** |  | **2021** | **EAPC** |
| Andean Latin America | 0.76 (0.6, 0.93) | −3.95 (−4.08, −3.83) |  | 74.89 (53.81, 106.06) | −1.84 (−1.96, −1.72) |  | 1122.6 (880.84, 1408.91) | 0.09 (0.07, 0.11) |  | 70.48 (55.05, 89.74) | −0.01 (−0.02, 0) |
| Australasia | 1.1 (0.94, 1.22) | −2.45 (−2.69, −2.21) |  | 26.93 (24.5, 29.26) | −2.65 (−2.87, −2.44) |  | 48.1 (40.98, 56.21) | −0.63 (−0.79, −0.46) |  | 3.74 (3.3, 4.25) | −1.05 (−1.23, −0.87) |
| Caribbean | 1.91 (1.49, 2.49) | −1.87 (−2, −1.74) |  | 141.67 (110.04, 182.02) | −1.23 (−1.34, −1.11) |  | 1117.59 (878.6, 1382.27) | 0.21 (0.2, 0.22) |  | 73.45 (57.79, 91.24) | 0.26 (0.25, 0.27) |
| Central Asia | 3.2 (2.79, 3.66) | −2.2 (−2.4, −1.99) |  | 142.06 (120.7, 167.98) | −2.3 (−2.48, −2.12) |  | 871.28 (685.87, 1081.67) | 0.14 (0.12, 0.15) |  | 56.67 (44.78, 71.18) | −0.03 (−0.08, 0.02) |
| Central Europe | 1.38 (1.2, 1.51) | −5.33 (−5.82, −4.83) |  | 34.96 (30.98, 38.82) | −5.91 (−6.4, −5.41) |  | 86.95 (77.16, 98.2) | −1.56 (−1.9, −1.22) |  | 6.49 (5.83, 7.19) | −2.31 (−2.5, −2.12) |
| Central Latin America | 0.48 (0.34, 0.56) | −6.06 (−6.24, −5.88) |  | 36.16 (26.76, 48.41) | −3.92 (−4.13, −3.71) |  | 479.91 (383.87, 587.9) | −0.1 (−0.16, −0.04) |  | 32.23 (25.7, 40.03) | −0.09 (−0.15, −0.03) |
| Central Sub−Saharan Africa | 4.11 (2.51, 6.89) | −2.14 (−2.25, −2.03) |  | 191.41 (135.53, 293.59) | −1.66 (−1.72, −1.59) |  | 1665.95 (1307.3, 2090.81) | −0.02 (−0.04, 0) |  | 116.22 (90.61, 147.07) | 0.02 (−0.02, 0.06) |
| East Asia | 4.15 (3.27, 5.26) | −4.97 (−5.11, −4.84) |  | 110.83 (90.18, 135.12) | −4.79 (−4.92, −4.66) |  | 614.54 (488.51, 756.55) | −0.21 (−0.36, −0.07) |  | 39.64 (31.4, 49.34) | −0.45 (−0.59, −0.31) |
| Eastern Europe | 1.04 (0.95, 1.15) | −5.95 (−6.36, −5.54) |  | 34.54 (31.06, 38.67) | −6.3 (−6.72, −5.88) |  | 150.14 (131.16, 171.17) | −1.77 (−1.86, −1.69) |  | 8.54 (7.52, 9.56) | −2.21 (−2.31, −2.11) |
| Eastern Sub−Saharan Africa | 3.48 (2.79, 4.19) | −2.51 (−2.59, −2.44) |  | 161.76 (129.27, 205.09) | −1.75 (−1.81, −1.69) |  | 1584.28 (1249.22, 1977.41) | 0.24 (0.22, 0.26) |  | 109.17 (84.65, 137.66) | 0.21 (0.19, 0.23) |
| High−income Asia Pacific | 0.75 (0.59, 0.85) | −3.45 (−3.53, −3.38) |  | 13.04 (11.25, 14.37) | −3.98 (−4.1, −3.87) |  | 33.31 (28.78, 38.14) | −1.56 (−1.65, −1.47) |  | 2.4 (2.12, 2.73) | −1.89 (−1.97, −1.8) |
| High−income North America | 0.85 (0.71, 0.93) | −3.26 (−3.81, −2.71) |  | 22.72 (19.91, 25.14) | −3.31 (−3.88, −2.74) |  | 122.33 (112.08, 134.26) | −0.91 (−1.28, −0.55) |  | 9.91 (9, 10.92) | −0.82 (−1.23, −0.4) |
| North Africa and Middle East | 1.9 (1.62, 2.23) | −3.11 (−3.21, −3) |  | 86.78 (71.3, 104.16) | −2.98 (−3.06, −2.9) |  | 540.77 (428.63, 660.44) | 0.18 (0.14, 0.22) |  | 37.29 (29.57, 46.07) | 0.21 (0.17, 0.25) |
| Oceania | 11.71 (7.03, 20.8) | −1.61 (−1.65, −1.56) |  | 525.51 (342.95, 854.77) | −1.3 (−1.35, −1.25) |  | 1000.63 (779.28, 1219.8) | 0.23 (0.18, 0.28) |  | 68.84 (54, 85.1) | 0.22 (0.17, 0.27) |
| South Asia | 14.88 (12.23, 20.01) | −2.01 (−2.13, −1.88) |  | 453.58 (380.1, 580.89) | −2.19 (−2.3, −2.07) |  | 732.2 (575.83, 916.47) | 0.2 (0.17, 0.24) |  | 51.33 (40.39, 64.3) | 0.16 (0.12, 0.2) |
| Southeast Asia | 1.08 (0.86, 1.39) | −3.48 (−3.54, −3.41) |  | 65.95 (53.38, 82.03) | −2.86 (−2.92, −2.81) |  | 409.86 (328.48, 501.01) | 0.13 (0.1, 0.17) |  | 28.17 (22.76, 34.68) | 0.03 (−0.01, 0.07) |
| Southern Latin America | 1.18 (1.03, 1.3) | −4.27 (−4.5, −4.04) |  | 55.94 (42.77, 73.71) | −2.9 (−2.99, −2.81) |  | 705.21 (560.2, 873.03) | 0.21 (0.18, 0.24) |  | 42.87 (33.68, 53.55) | 0.16 (0.13, 0.2) |
| Southern Sub−Saharan Africa | 2.49 (2.08, 2.95) | −1.31 (−1.7, −0.91) |  | 165.06 (132.26, 208.39) | −0.81 (−1.08, −0.54) |  | 1506.2 (1189.06, 1867.03) | 0.01 (0.01, 0.02) |  | 99.22 (77.31, 125.64) | 0 (−0.01, 0) |
| Tropical Latin America | 1.09 (0.94, 1.17) | −2.86 (−3.02, −2.7) |  | 94.88 (71.08, 126.96) | −1.63 (−1.7, −1.56) |  | 1266.24 (1005.05, 1572.84) | 0 (−0.01, 0.01) |  | 78.98 (61.26, 99.83) | −0.06 (−0.07, −0.05) |
| Western Europe | 1.51 (1.27, 1.65) | −2.19 (−2.37, −2.02) |  | 28.17 (25.48, 30.3) | −2.76 (−2.98, −2.55) |  | 43.87 (38.06, 51.01) | −1.14 (−1.19, −1.1) |  | 4.37 (3.95, 4.84) | −1.15 (−1.18, −1.12) |
| Western Sub−Saharan Africa | 2.17 (1.8, 2.69) | −4.02 (−4.21, −3.83) |  | 116.95 (89.91, 149.81) | −2.79 (−2.97, −2.61) |  | 1148.61 (895.18, 1426.16) | 0.21 (0.19, 0.23) |  | 78.59 (61.43, 98.68) | 0.18 (0.16, 0.2) |

**Supplemental Table 2.** **Age -standardized rates and estimated annual percentage change for rheumatic heart disease burden (1990–2021) across 204 countries and territories**

|  | **ASMR (95% UI)** | |  | **ASDR (95% UI)** | |  | **ASPR (95% UI)** | |  | **ASIR (95% UI)** | |
| --- | --- | --- | --- | --- | --- | --- | --- | --- | --- | --- | --- |
|  | **2021** | **EAPC** |  | **2021** | **EAPC** |  | **2021** | **EAPC** |  | **2021** | **EAPC** |
| Afghanistan | 4.18 (2.49, 6.58) | −2.54 (−2.74, −2.33) |  | 174.77 (116.97, 247.14) | −2.45 (−2.63, −2.27) |  | 698.19 (550.94, 858.63) | 0.03 (0.03, 0.04) |  | 52.76 (41.79, 65.57) | 0.01 (0, 0.03) |
| Albania | 1.39 (0.87, 1.96) | −4.53 (−5.01, −4.04) |  | 85.01 (63.08, 115.06) | −2.76 (−3.08, −2.44) |  | 1076.53 (841.3, 1332.79) | −0.06 (−0.08, −0.03) |  | 62.59 (48.68, 79.26) | −0.23 (−0.24, −0.21) |
| Algeria | 1.4 (1.09, 1.78) | −2.17 (−2.31, −2.03) |  | 65.25 (50.29, 83.85) | −2.08 (−2.16, −2) |  | 574.42 (451.13, 711.4) | 0.3 (0.19, 0.41) |  | 37.27 (29.36, 46.82) | 0.26 (0.15, 0.37) |
| American Samoa | 5.27 (4.1, 6.61) | −1.46 (−1.54, −1.39) |  | 245.53 (196.64, 298.82) | −1.05 (−1.1, −1.01) |  | 1123.51 (886.47, 1376.69) | 0.1 (0.09, 0.12) |  | 68.97 (54.08, 85.85) | 0.16 (0.14, 0.17) |
| Andorra | 0.57 (0.4, 0.75) | −2.04 (−2.23, −1.85) |  | 12.68 (9.3, 16.52) | −2.17 (−2.33, −2) |  | 35.47 (29.46, 42.32) | −0.73 (−0.85, −0.61) |  | 3.12 (2.73, 3.59) | −0.93 (−1.09, −0.76) |
| Angola | 4.25 (2.85, 6.44) | −2.86 (−2.95, −2.77) |  | 195.96 (139.28, 268.89) | −2.26 (−2.32, −2.2) |  | 1643.36 (1289.62, 2062.81) | 0.01 (0, 0.02) |  | 110.54 (85.99, 138.6) | −0.06 (−0.08, −0.04) |
| Antigua and Barbuda | 0.69 (0.63, 0.75) | −2.86 (−3.16, −2.55) |  | 82.07 (59.36, 113.32) | −1.31 (−1.44, −1.18) |  | 1212.66 (961.71, 1501.85) | 0.03 (0.02, 0.04) |  | 68.05 (52.84, 86.41) | −0.02 (−0.04, 0) |
| Argentina | 1.46 (1.27, 1.62) | −3.84 (−4.07, −3.62) |  | 74.73 (56.38, 100.46) | −2.37 (−2.47, −2.27) |  | 1014.45 (803.02, 1264.51) | 0.17 (0.13, 0.2) |  | 59.86 (46.6, 75.31) | 0.07 (0.04, 0.1) |
| Armenia | 1.89 (1.36, 2.32) | −2.56 (−3.01, −2.1) |  | 104.18 (77, 135.03) | −2.24 (−2.45, −2.02) |  | 1114.26 (879.67, 1368.22) | −0.19 (−0.21, −0.18) |  | 64.21 (49.81, 79.22) | −0.31 (−0.36, −0.27) |
| Australia | 0.99 (0.84, 1.1) | −2.2 (−2.62, −1.79) |  | 23.5 (21.16, 25.57) | −2.41 (−2.75, −2.06) |  | 46.54 (38.61, 55.19) | −0.68 (−0.87, −0.5) |  | 3.51 (3.05, 4.02) | −1.04 (−1.26, −0.81) |
| Austria | 1.74 (1.47, 1.92) | −1.62 (−2.07, −1.16) |  | 31.38 (27.9, 34.11) | −2.67 (−3.11, −2.22) |  | 58.2 (52.78, 64.9) | −5.32 (−5.73, −4.92) |  | 5.1 (4.71, 5.55) | −4.17 (−4.54, −3.81) |
| Azerbaijan | 1.38 (0.83, 1.99) | −2.27 (−2.65, −1.88) |  | 93.75 (68.44, 125.14) | −1.68 (−1.91, −1.45) |  | 1029.48 (810.99, 1288.04) | −0.11 (−0.12, −0.1) |  | 63.13 (49.45, 80.24) | −0.24 (−0.29, −0.18) |
| Bahamas | 0.63 (0.49, 0.8) | −2.71 (−2.95, −2.47) |  | 81.46 (58.51, 111.35) | −1.15 (−1.25, −1.05) |  | 1175.01 (925.83, 1468.96) | 0.05 (0.05, 0.06) |  | 71.55 (55.06, 90.52) | 0.05 (0.03, 0.06) |
| Bahrain | 0.75 (0.6, 0.94) | −6.06 (−6.57, −5.54) |  | 20.58 (16.49, 25.44) | −6.09 (−6.65, −5.53) |  | 59.95 (49.65, 70.87) | −0.81 (−0.87, −0.74) |  | 4.36 (3.73, 5.07) | −0.99 (−1.07, −0.91) |
| Bangladesh | 16.31 (11.15, 27.91) | −1.91 (−2.17, −1.65) |  | 457.57 (324.99, 745.57) | −2.08 (−2.24, −1.93) |  | 755.64 (588.79, 937.02) | 0.19 (0.16, 0.21) |  | 51.47 (39.54, 65.17) | 0.09 (0.07, 0.11) |
| Barbados | 0.59 (0.45, 0.77) | −2.88 (−3.14, −2.63) |  | 79.56 (56.56, 109.51) | −1.17 (−1.26, −1.07) |  | 1210.66 (950.51, 1502.5) | 0.12 (0.1, 0.14) |  | 71.13 (55.44, 89.81) | 0.12 (0.09, 0.16) |
| Belarus | 1.25 (1.02, 1.52) | −3.62 (−4.39, −2.84) |  | 44.19 (36.09, 53.78) | −3.55 (−4.29, −2.8) |  | 156.93 (132.45, 187.15) | −1.77 (−1.84, −1.69) |  | 8.68 (7.49, 10.09) | −2.29 (−2.33, −2.24) |
| Belgium | 1.49 (1.24, 1.67) | −0.61 (−0.94, −0.27) |  | 27.43 (24.1, 30.22) | −1.05 (−1.3, −0.8) |  | 46.04 (38.14, 55.87) | −0.2 (−1.08, 0.68) |  | 4.51 (3.94, 5.16) | 0.49 (−0.15, 1.15) |
| Belize | 0.69 (0.6, 0.79) | −2.36 (−2.81, −1.91) |  | 83.17 (62.26, 111.81) | −1.12 (−1.32, −0.93) |  | 1163.65 (926.09, 1433.83) | 0.07 (0.06, 0.08) |  | 73.97 (57.83, 94.29) | 0.01 (−0.01, 0.02) |
| Benin | 2.02 (1.44, 2.71) | −3.47 (−3.78, −3.17) |  | 109 (81.72, 143.63) | −2.31 (−2.57, −2.04) |  | 1096.13 (861.05, 1352.64) | 0.12 (0.08, 0.15) |  | 76.26 (59.16, 95.75) | 0.1 (0.07, 0.14) |
| Bermuda | 0.39 (0.32, 0.49) | −4.03 (−4.39, −3.66) |  | 16.27 (13.7, 19.58) | −3.91 (−4.25, −3.57) |  | 46.19 (38.23, 54.89) | −1.39 (−1.54, −1.23) |  | 2.95 (2.46, 3.49) | −1.56 (−1.72, −1.41) |
| Bhutan | 14.26 (8.04, 27.41) | −2.59 (−2.68, −2.5) |  | 401.91 (251.17, 725.13) | −2.93 (−3.01, −2.84) |  | 733.16 (576.89, 916.42) | 0.04 (0.03, 0.06) |  | 47.93 (37.58, 60.27) | −0.04 (−0.06, −0.03) |
| Bolivia (Plurinational State of) | 1.7 (1.26, 2.35) | −3.46 (−3.62, −3.3) |  | 98.78 (72.78, 134.15) | −2.39 (−2.54, −2.23) |  | 1124.81 (875.96, 1422.41) | 0 (−0.01, 0.01) |  | 74.21 (58.16, 94.36) | −0.08 (−0.09, −0.07) |
| Bosnia and Herzegovina | 0.7 (0.52, 0.92) | −4.93 (−5.38, −4.47) |  | 18.63 (14.3, 24.2) | −5.04 (−5.48, −4.6) |  | 44.53 (36.09, 53.14) | −0.49 (−0.66, −0.33) |  | 3.25 (2.74, 3.82) | −1.65 (−1.89, −1.41) |
| Botswana | 1.59 (1.11, 2.23) | −3.7 (−3.96, −3.44) |  | 120.35 (87.81, 164.92) | −2.21 (−2.4, −2.01) |  | 1427.13 (1114.54, 1758.43) | −0.04 (−0.05, −0.04) |  | 91.45 (71.63, 113.95) | −0.12 (−0.13, −0.1) |
| Brazil | 1.09 (0.95, 1.18) | −2.88 (−3.04, −2.72) |  | 95.14 (71.26, 127.27) | −1.64 (−1.71, −1.57) |  | 1268.37 (1006.92, 1575.54) | 0 (−0.01, 0.02) |  | 79.25 (61.47, 100.29) | −0.06 (−0.07, −0.04) |
| Brunei Darussalam | 2.1 (1.74, 2.48) | −1.67 (−1.9, −1.44) |  | 44.13 (37.57, 51.38) | −2.14 (−2.33, −1.95) |  | 70.13 (58.34, 83.2) | −1.02 (−1.11, −0.92) |  | 5.49 (4.83, 6.31) | −1.09 (−1.23, −0.94) |
| Bulgaria | 2.01 (1.57, 2.49) | −4.63 (−5.18, −4.07) |  | 60.77 (47.16, 74.8) | −4.67 (−5.24, −4.1) |  | 57.71 (49.2, 67.71) | −1.68 (−1.97, −1.39) |  | 4.59 (4.05, 5.22) | −2.78 (−3.09, −2.46) |
| Burkina Faso | 4.09 (2.78, 5.98) | −2.36 (−2.65, −2.07) |  | 158.45 (115.46, 218.19) | −1.97 (−2.23, −1.72) |  | 1046.9 (806.85, 1322.75) | 0.01 (0, 0.02) |  | 75.16 (57.37, 95.38) | 0 (−0.01, 0.01) |
| Burundi | 3.98 (2.8, 5.71) | −3.41 (−3.66, −3.16) |  | 176.12 (128.35, 235.33) | −2.58 (−2.79, −2.38) |  | 1593.99 (1250.98, 1985.76) | 0.17 (0.16, 0.17) |  | 115.2 (88.48, 147.03) | 0.22 (0.2, 0.23) |
| Cabo Verde | 1.26 (0.95, 1.65) | −5.17 (−6.04, −4.3) |  | 88.88 (66.37, 118.49) | −3.14 (−3.7, −2.56) |  | 1159.04 (899.48, 1434.86) | 0.01 (−0.01, 0.03) |  | 71.5 (55.75, 89.97) | −0.09 (−0.11, −0.06) |
| Cambodia | 1.56 (1.04, 2.14) | −4.03 (−4.2, −3.86) |  | 94.91 (67.98, 127.01) | −3.46 (−3.62, −3.29) |  | 681.89 (534.69, 853.69) | −0.22 (−0.3, −0.14) |  | 48.8 (38.45, 61.02) | −0.29 (−0.36, −0.21) |
| Cameroon | 2.21 (1.57, 3.16) | −3.5 (−3.59, −3.41) |  | 113.58 (82.93, 152.08) | −2.29 (−2.35, −2.23) |  | 1092.37 (850.65, 1366.77) | 0.23 (0.17, 0.29) |  | 76.34 (59.49, 96.57) | 0.24 (0.18, 0.3) |
| Canada | 0.99 (0.83, 1.08) | −2.41 (−2.88, −1.94) |  | 23.88 (21.27, 26.76) | −2.56 (−2.99, −2.14) |  | 114.13 (93.81, 134.81) | −0.66 (−0.75, −0.57) |  | 8.87 (7.31, 10.53) | −0.71 (−0.8, −0.62) |
| Central African Republic | 6.87 (3.58, 12.3) | −1.8 (−1.89, −1.7) |  | 278.72 (176.39, 450.58) | −1.5 (−1.57, −1.43) |  | 1668.92 (1302.75, 2101.17) | 0.05 (0.04, 0.06) |  | 122.8 (95.59, 154.64) | 0.09 (0.08, 0.11) |
| Chad | 3.99 (2.74, 5.39) | −2.38 (−2.48, −2.28) |  | 167.06 (122.5, 215.76) | −1.81 (−1.9, −1.72) |  | 1054.07 (814.92, 1325.18) | 0.03 (0.02, 0.05) |  | 76.02 (59.45, 98.74) | 0.01 (0, 0.02) |
| Chile | 0.71 (0.61, 0.79) | −5.91 (−6.2, −5.63) |  | 18.23 (16.04, 20.48) | −6.15 (−6.39, −5.9) |  | 57.91 (48.03, 69.24) | −2.02 (−2.16, −1.88) |  | 3.97 (3.41, 4.67) | −2.8 (−2.96, −2.64) |
| China | 4.21 (3.3, 5.37) | −5 (−5.14, −4.86) |  | 111.69 (90.42, 136.81) | −4.83 (−4.97, −4.7) |  | 619.85 (492.05, 763.74) | −0.21 (−0.36, −0.06) |  | 39.86 (31.52, 49.65) | −0.46 (−0.61, −0.32) |
| Colombia | 0.2 (0.15, 0.25) | −8.05 (−8.63, −7.45) |  | 8.31 (6.65, 10.03) | −7.3 (−7.74, −6.86) |  | 45.36 (37.48, 53.26) | −1.04 (−1.29, −0.79) |  | 2.98 (2.48, 3.54) | −1.66 (−1.94, −1.39) |
| Comoros | 2.96 (2.13, 3.89) | −2.52 (−2.69, −2.34) |  | 148.41 (109.71, 194.05) | −1.71 (−1.87, −1.56) |  | 1610.76 (1264.05, 2041.42) | 0.1 (0.09, 0.11) |  | 108.46 (83.96, 137.67) | 0.14 (0.12, 0.16) |
| Congo | 3.7 (2.57, 5.17) | −3.28 (−3.45, −3.11) |  | 180.72 (131.24, 238.93) | −2.45 (−2.59, −2.3) |  | 1677.7 (1303.4, 2094.76) | −0.2 (−0.31, −0.08) |  | 113.31 (88.14, 142.51) | −0.21 (−0.32, −0.09) |
| Cook Islands | 1.66 (1.28, 2.07) | −2.49 (−2.65, −2.33) |  | 67.84 (52.62, 85.05) | −2.33 (−2.52, −2.15) |  | 123.78 (105.15, 144.44) | −0.12 (−0.24, 0) |  | 7.94 (6.99, 9.08) | −0.51 (−0.64, −0.38) |
| Costa Rica | 0.58 (0.5, 0.66) | −5.86 (−6.11, −5.61) |  | 78.56 (54.6, 110.83) | −2.35 (−2.48, −2.22) |  | 1290.11 (1030.51, 1574.55) | 0.04 (0.03, 0.04) |  | 72.26 (55.95, 90.42) | −0.07 (−0.08, −0.06) |
| Côte d'Ivoire | 2.24 (1.7, 2.96) | −3.1 (−3.25, −2.96) |  | 116.39 (87.34, 152.77) | −2.01 (−2.14, −1.88) |  | 1102.19 (854.39, 1379.24) | 0.06 (0.05, 0.07) |  | 76.19 (59.3, 96.36) | 0.09 (0.07, 0.11) |
| Croatia | 1.27 (1.08, 1.48) | −4.33 (−5.25, −3.4) |  | 27.86 (23.73, 32.16) | −5.02 (−5.78, −4.26) |  | 45.32 (39.49, 51.71) | −1.99 (−2.29, −1.69) |  | 3.94 (3.58, 4.35) | −2.16 (−2.59, −1.74) |
| Cuba | 1.07 (0.93, 1.22) | −2.76 (−3.08, −2.44) |  | 95.03 (72.32, 126.59) | −1.76 (−1.92, −1.6) |  | 1188.83 (941.05, 1474.39) | −0.07 (−0.09, −0.05) |  | 69.83 (53.98, 89.07) | −0.11 (−0.15, −0.08) |
| Cyprus | 2.65 (2.13, 3.14) | −3.72 (−4.05, −3.39) |  | 44.06 (36.74, 51.44) | −3.85 (−4.09, −3.61) |  | 34.68 (28.29, 42.38) | −2.14 (−2.62, −1.65) |  | 4.89 (4.37, 5.46) | −2.46 (−2.82, −2.11) |
| Czechia | 1.32 (1.13, 1.52) | −4.34 (−5.09, −3.6) |  | 29.39 (25.54, 33.45) | −5.25 (−5.95, −4.55) |  | 62.48 (52.01, 74.96) | −1.2 (−1.52, −0.87) |  | 5.06 (4.4, 5.8) | −1.82 (−2.2, −1.45) |
| Democratic People's Republic of Korea | 5.89 (3.4, 11.2) | −2.21 (−2.32, −2.1) |  | 169.1 (108.61, 289.76) | −2.02 (−2.08, −1.96) |  | 710.71 (553.19, 879.04) | −0.31 (−0.34, −0.27) |  | 48.63 (38.16, 60.74) | −0.22 (−0.25, −0.19) |
| Democratic Republic of the Congo | 4.02 (2.12, 7.75) | −1.79 (−1.93, −1.64) |  | 187.64 (125.43, 311.41) | −1.38 (−1.46, −1.29) |  | 1674.4 (1306.42, 2111.01) | −0.02 (−0.05, 0.01) |  | 118.5 (91.98, 150.8) | 0.07 (0.01, 0.12) |
| Denmark | 1.11 (0.95, 1.22) | −0.87 (−1.2, −0.55) |  | 20.85 (18.98, 22.48) | −1.76 (−2.02, −1.5) |  | 21.61 (17.14, 26.29) | −2 (−2.91, −1.07) |  | 2.04 (1.74, 2.36) | −2.46 (−3.49, −1.43) |
| Djibouti | 2.79 (1.8, 4.08) | −2.52 (−2.63, −2.42) |  | 142.44 (103.83, 194.43) | −1.59 (−1.67, −1.51) |  | 1591.38 (1259.92, 1998.38) | 0.17 (0.16, 0.19) |  | 105.15 (81.82, 132.3) | 0.18 (0.16, 0.19) |
| Dominica | 1.34 (1.06, 1.66) | −2.24 (−2.44, −2.04) |  | 107.34 (81.11, 140.68) | −1.15 (−1.31, −0.99) |  | 1190.02 (936.09, 1474.46) | −0.01 (−0.03, 0.01) |  | 74.68 (57.85, 93.59) | −0.04 (−0.05, −0.03) |
| Dominican Republic | 0.99 (0.71, 1.49) | −2.07 (−2.23, −1.91) |  | 98.42 (72.73, 131.05) | −1.2 (−1.33, −1.08) |  | 1140.61 (908.51, 1410.97) | 0.03 (0.02, 0.03) |  | 71 (55.84, 88.38) | −0.1 (−0.11, −0.09) |
| Ecuador | 0.64 (0.45, 0.81) | −4.33 (−4.77, −3.9) |  | 69.73 (47.91, 98.72) | −2.08 (−2.27, −1.89) |  | 1103.97 (854.01, 1378.44) | 0.08 (0.06, 0.09) |  | 70.1 (54.4, 89.78) | 0.01 (0, 0.02) |
| Egypt | 4.37 (3.59, 5.3) | −3.16 (−3.25, −3.07) |  | 172.85 (143.5, 206.22) | −3.5 (−3.61, −3.38) |  | 823.47 (640.14, 1006.86) | 0.28 (0.22, 0.34) |  | 52.4 (40.9, 65.33) | 0.16 (0.1, 0.22) |
| El Salvador | 0.3 (0.23, 0.4) | −4.62 (−5, −4.25) |  | 65.06 (44.18, 94.86) | −1.2 (−1.36, −1.05) |  | 1166.07 (918.94, 1440.93) | 0.06 (0.05, 0.07) |  | 75.69 (59.23, 95.68) | −0.04 (−0.05, −0.03) |
| Equatorial Guinea | 1.94 (1.22, 2.99) | −6.33 (−6.85, −5.82) |  | 129.5 (89.7, 181.17) | −4.14 (−4.56, −3.73) |  | 1638.51 (1282.07, 2035.97) | −0.11 (−0.15, −0.08) |  | 103.15 (79.86, 130.34) | −0.48 (−0.58, −0.37) |
| Eritrea | 5.16 (3.72, 6.64) | −2.35 (−2.49, −2.21) |  | 215.11 (161.77, 271.17) | −1.84 (−1.96, −1.71) |  | 1865.41 (1480.34, 2332.94) | 0.13 (0, 0.25) |  | 130.35 (101.92, 163.49) | 0.13 (0.01, 0.24) |
| Estonia | 0.63 (0.54, 0.73) | −7.77 (−8.22, −7.32) |  | 23.96 (20.33, 28.3) | −7.58 (−8.04, −7.12) |  | 152.34 (125.29, 185.28) | −1.82 (−1.96, −1.69) |  | 7.97 (6.68, 9.49) | −2.37 (−2.52, −2.22) |
| Eswatini | 2.79 (1.9, 3.93) | −1.99 (−2.42, −1.57) |  | 163.85 (119.2, 216.88) | −1.27 (−1.59, −0.95) |  | 1405.13 (1097.7, 1766.01) | 0.01 (0, 0.02) |  | 95.05 (73.58, 120.75) | 0 (−0.02, 0.01) |
| Ethiopia | 2.92 (2.05, 4.26) | −4.08 (−4.29, −3.86) |  | 144.92 (109.37, 193.7) | −2.85 (−2.98, −2.71) |  | 1524.08 (1194.62, 1913.92) | 0.54 (0.48, 0.6) |  | 105.74 (81.22, 134.09) | 0.49 (0.43, 0.55) |
| Fiji | 8.83 (6.79, 11.33) | −2.27 (−2.38, −2.16) |  | 371.12 (287.18, 474.31) | −1.96 (−2.03, −1.88) |  | 835.46 (664.6, 1030.22) | 1.21 (0.84, 1.59) |  | 54.89 (43.36, 68.16) | 1.16 (0.78, 1.54) |
| Finland | 0.71 (0.61, 0.78) | −2.97 (−3.1, −2.85) |  | 16.34 (14.88, 17.78) | −2.96 (−3.11, −2.8) |  | 17.44 (14.17, 20.98) | −3.72 (−4.55, −2.89) |  | 1.43 (1.2, 1.66) | −4.11 (−4.64, −3.59) |
| France | 1.23 (1.02, 1.39) | −2.44 (−2.87, −2.01) |  | 23.75 (20.9, 26.41) | −2.55 (−2.89, −2.21) |  | 35.19 (28.89, 42.47) | −1.04 (−1.13, −0.95) |  | 3.61 (3.16, 4.11) | −1.24 (−1.39, −1.08) |
| Gabon | 2.46 (1.8, 3.33) | −3.2 (−3.34, −3.06) |  | 140.81 (105.74, 189.6) | −1.99 (−2.06, −1.92) |  | 1619.98 (1270.78, 2019.6) | 0.05 (0.04, 0.07) |  | 102.13 (78.97, 129.95) | 0.09 (0.08, 0.1) |
| Gambia | 2.54 (1.91, 3.53) | −3.01 (−3.37, −2.64) |  | 126.09 (95.78, 170.2) | −2.05 (−2.32, −1.77) |  | 1136.3 (883.92, 1432.16) | 0.17 (0.15, 0.18) |  | 79.2 (61.95, 101.23) | 0.19 (0.18, 0.21) |
| Georgia | 4.32 (3.69, 5.07) | −0.77 (−1.35, −0.18) |  | 169.28 (142.89, 201.81) | −1.84 (−2.25, −1.42) |  | 1078.73 (847.53, 1331.07) | −0.03 (−0.07, 0) |  | 65.53 (51.15, 81.99) | −0.08 (−0.1, −0.05) |
| Germany | 2.46 (2.06, 2.73) | −0.88 (−1.33, −0.43) |  | 41.81 (36.93, 45.51) | −1.68 (−2.21, −1.15) |  | 38.99 (32.34, 46.76) | −0.86 (−1.1, −0.62) |  | 4.43 (3.93, 4.97) | −0.84 (−0.98, −0.71) |
| Ghana | 1.36 (1.03, 1.77) | −4.7 (−5.08, −4.32) |  | 88.14 (65.91, 117.25) | −2.92 (−3.17, −2.67) |  | 1076.01 (841.63, 1349.56) | 0 (−0.02, 0.02) |  | 73.34 (57.62, 92.75) | −0.05 (−0.07, −0.03) |
| Greece | 0.68 (0.6, 0.75) | −1.4 (−2.23, −0.56) |  | 13.91 (12.43, 15.18) | −1.34 (−2.02, −0.66) |  | 27.3 (22.59, 32.19) | −1 (−1.22, −0.79) |  | 2.2 (1.91, 2.52) | −1.76 (−2.1, −1.42) |
| Greenland | 1.02 (0.78, 1.26) | −5.42 (−5.77, −5.07) |  | 27.38 (22.59, 32.33) | −4.86 (−5.17, −4.54) |  | 137.88 (113.84, 164.69) | −1.39 (−1.48, −1.31) |  | 10.73 (8.88, 12.96) | −1.81 (−1.93, −1.7) |
| Grenada | 2.18 (1.87, 2.5) | −2.55 (−2.91, −2.19) |  | 137.86 (112.32, 173.46) | −1.94 (−2.2, −1.69) |  | 1187.48 (933.72, 1475.08) | −0.02 (−0.02, −0.01) |  | 73.25 (56.93, 93.45) | −0.12 (−0.13, −0.11) |
| Guam | 1.77 (1.52, 2.05) | −2.05 (−2.5, −1.61) |  | 127.78 (104.65, 158.77) | −0.77 (−1.04, −0.5) |  | 1066.83 (835.1, 1308.69) | 0.17 (0.16, 0.19) |  | 61.59 (48.26, 76.74) | 0.14 (0.13, 0.15) |
| Guatemala | 0.16 (0.13, 0.19) | −7.07 (−7.35, −6.79) |  | 59.75 (38.88, 88.33) | −1.43 (−1.66, −1.21) |  | 1158.51 (919.12, 1420.63) | 0.34 (0.3, 0.39) |  | 79.53 (62.17, 99.9) | 0.26 (0.22, 0.3) |
| Guinea | 3.07 (2.11, 4.36) | −3.22 (−3.44, −3.01) |  | 132.91 (97.52, 177.88) | −2.54 (−2.74, −2.34) |  | 904.3 (703.16, 1121.8) | 0.24 (0.19, 0.29) |  | 65.8 (51.33, 82.6) | 0.25 (0.2, 0.31) |
| Guinea−Bissau | 4.11 (2.92, 5.71) | −3.33 (−3.56, −3.1) |  | 173.49 (132.44, 231.14) | −2.8 (−3, −2.6) |  | 1094.82 (848.42, 1384.06) | 0.13 (0.11, 0.14) |  | 80.57 (62.94, 102.75) | 0.14 (0.12, 0.16) |
| Guyana | 1.37 (1.01, 1.83) | −2.32 (−2.59, −2.04) |  | 112.06 (87.31, 145.63) | −1.43 (−1.61, −1.26) |  | 1157.46 (907.51, 1428.22) | 0.02 (0, 0.03) |  | 79.06 (62.18, 99.71) | −0.06 (−0.08, −0.05) |
| Haiti | 5.01 (2.93, 7.93) | −2.26 (−2.37, −2.16) |  | 279.83 (179.47, 408.93) | −2.01 (−2.14, −1.89) |  | 1245.29 (972.24, 1551.07) | 0.16 (0.15, 0.18) |  | 90.03 (70.67, 111.93) | 0.15 (0.13, 0.17) |
| Honduras | 0.71 (0.41, 1.08) | −1.16 (−1.33, −0.99) |  | 75.76 (51.65, 105.5) | −0.53 (−0.57, −0.48) |  | 1175.78 (918.94, 1463.5) | 0.05 (0.03, 0.06) |  | 78.73 (60.74, 99.4) | 0.01 (−0.01, 0.02) |
| Hungary | 1.17 (1.01, 1.34) | −5.7 (−6.41, −4.99) |  | 28.52 (24.65, 32.38) | −6.35 (−7.04, −5.66) |  | 64.18 (53.14, 76.24) | −2.22 (−2.62, −1.83) |  | 5.1 (4.44, 5.86) | −3.02 (−3.46, −2.57) |
| Iceland | 0.65 (0.54, 0.74) | −1.34 (−1.59, −1.09) |  | 13.84 (12.13, 15.51) | −1.58 (−1.87, −1.29) |  | 22.04 (18.21, 26.55) | −0.99 (−1.12, −0.86) |  | 1.83 (1.58, 2.12) | −1.34 (−1.49, −1.2) |
| India | 14.28 (11.79, 19) | −2.04 (−2.2, −1.88) |  | 431.47 (361.64, 548.57) | −2.32 (−2.46, −2.18) |  | 690.86 (539.59, 867.33) | 0.15 (0.09, 0.21) |  | 48.31 (37.83, 61.05) | 0.1 (0.03, 0.16) |
| Indonesia | 1.31 (0.94, 1.94) | −3.21 (−3.31, −3.11) |  | 62.46 (47.39, 86.27) | −3.17 (−3.25, −3.09) |  | 206.73 (171.83, 248.4) | 0.3 (0.26, 0.35) |  | 15.43 (12.88, 18.62) | −0.1 (−0.12, −0.08) |
| Iran (Islamic Republic of) | 1.38 (1.12, 1.59) | −2.83 (−3.19, −2.47) |  | 69.8 (55.69, 88.53) | −2.26 (−2.5, −2.02) |  | 709.91 (558.26, 877.39) | 0.07 (0.05, 0.08) |  | 44.44 (34.94, 55.67) | −0.03 (−0.05, −0.01) |
| Iraq | 1.87 (1.4, 2.39) | −3.05 (−3.2, −2.89) |  | 88.37 (69.29, 110.57) | −2.58 (−2.71, −2.46) |  | 741.76 (575.05, 921) | 0.05 (0.03, 0.06) |  | 47.89 (37.74, 59.48) | −0.05 (−0.08, −0.02) |
| Ireland | 0.75 (0.63, 0.86) | −2.87 (−3.09, −2.65) |  | 15.69 (13.43, 17.63) | −3.08 (−3.27, −2.89) |  | 30.58 (25.32, 36.71) | −1.45 (−1.62, −1.28) |  | 2.61 (2.25, 3) | −1.88 (−2.09, −1.67) |
| Israel | 1.47 (1.28, 1.63) | −2.01 (−2.24, −1.78) |  | 29.34 (26.56, 31.95) | −2.4 (−2.58, −2.21) |  | 39.14 (32.31, 47.51) | −0.96 (−1.01, −0.92) |  | 3.79 (3.32, 4.34) | −0.97 (−1.03, −0.9) |
| Italy | 1.6 (1.36, 1.77) | −1.87 (−2.13, −1.61) |  | 33.18 (29.99, 35.97) | −2.61 (−2.86, −2.35) |  | 68.61 (62.44, 75.68) | −0.93 (−1.23, −0.62) |  | 6.05 (5.48, 6.68) | −0.81 (−0.97, −0.65) |
| Jamaica | 1.09 (0.81, 1.44) | −2.57 (−2.9, −2.25) |  | 100.52 (76.45, 132.84) | −1.44 (−1.64, −1.25) |  | 1208.39 (948.53, 1489.12) | 0.18 (0.16, 0.2) |  | 70.75 (54.21, 89.51) | 0.13 (0.12, 0.15) |
| Japan | 0.79 (0.62, 0.89) | −3.4 (−3.49, −3.3) |  | 13.86 (12, 15.19) | −3.89 (−4.03, −3.75) |  | 35.73 (31.23, 40.55) | −1.53 (−1.64, −1.41) |  | 2.69 (2.36, 3.07) | −1.74 (−1.84, −1.64) |
| Jordan | 0.47 (0.35, 0.59) | −3.46 (−3.72, −3.2) |  | 16.74 (13.21, 20.59) | −3.63 (−3.87, −3.38) |  | 41.42 (34.61, 48.94) | −0.71 (−0.79, −0.64) |  | 2.7 (2.29, 3.15) | −1.14 (−1.2, −1.07) |
| Kazakhstan | 1.9 (1.46, 2.35) | −5.68 (−6.11, −5.26) |  | 58.05 (44.39, 70.86) | −6.46 (−6.96, −5.95) |  | 107.96 (92.05, 126.22) | −1.9 (−1.99, −1.81) |  | 7.18 (6.36, 8.14) | −2.79 (−2.94, −2.64) |
| Kenya | 2.26 (1.45, 3.31) | −1 (−1.19, −0.81) |  | 121.02 (88.66, 160.92) | −0.56 (−0.67, −0.45) |  | 1489.91 (1173.67, 1851.62) | 0.13 (0.1, 0.15) |  | 98.27 (76.67, 124.44) | 0.13 (0.1, 0.16) |
| Kiribati | 11.59 (8.58, 15.86) | −1.24 (−1.32, −1.15) |  | 502.36 (378.27, 678.55) | −1.21 (−1.29, −1.14) |  | 1034.38 (808.65, 1276.64) | 0.07 (0.06, 0.08) |  | 74.8 (58.84, 93.4) | 0.08 (0.07, 0.08) |
| Kuwait | 0.39 (0.29, 0.48) | −6.14 (−7.59, −4.68) |  | 15.32 (12.4, 18.44) | −5.99 (−7.27, −4.7) |  | 50.51 (42.18, 58.82) | −0.84 (−0.91, −0.77) |  | 3.19 (2.66, 3.75) | −1.15 (−1.21, −1.09) |
| Kyrgyzstan | 2.89 (2.35, 3.59) | −3.5 (−4.02, −2.98) |  | 143 (117.19, 175.22) | −3.25 (−3.69, −2.8) |  | 1003.98 (789.42, 1241.1) | −0.1 (−0.11, −0.08) |  | 66.35 (51.8, 83.25) | −0.06 (−0.1, −0.02) |
| Lao People's Democratic Republic | 2.32 (1.45, 3.79) | −3.95 (−4.04, −3.86) |  | 147.88 (104.04, 220.2) | −3.27 (−3.33, −3.21) |  | 889.23 (687.05, 1114.02) | −0.07 (−0.22, 0.09) |  | 62.09 (48.36, 78.21) | −0.17 (−0.32, −0.01) |
| Latvia | 1.03 (0.89, 1.18) | −6.35 (−6.82, −5.88) |  | 37.27 (32.5, 43.27) | −6.22 (−6.69, −5.75) |  | 167.84 (141.53, 200.64) | −1.88 (−2.17, −1.59) |  | 8.81 (7.39, 10.37) | −2.46 (−2.64, −2.27) |
| Lebanon | 1.07 (0.89, 1.3) | −5.41 (−5.71, −5.1) |  | 32.6 (27.62, 39.04) | −5.29 (−5.61, −4.97) |  | 49.61 (41.28, 58.14) | −0.6 (−0.68, −0.53) |  | 3.46 (2.92, 4.07) | −0.91 (−0.98, −0.85) |
| Lesotho | 3.74 (2.42, 5.65) | −0.1 (−0.51, 0.3) |  | 195.31 (139.96, 271.52) | 0.14 (−0.17, 0.45) |  | 1415.38 (1103.11, 1767.19) | −0.01 (−0.02, 0) |  | 99.07 (76.96, 125.6) | −0.04 (−0.05, −0.03) |
| Liberia | 2.57 (1.8, 4.08) | −3.67 (−3.93, −3.41) |  | 125.97 (93.73, 182.33) | −2.69 (−2.97, −2.42) |  | 1117.1 (880.71, 1387) | 0.13 (0.11, 0.14) |  | 80.29 (62.63, 100.39) | 0.13 (0.11, 0.16) |
| Libya | 1.36 (0.88, 1.98) | −1.42 (−1.69, −1.15) |  | 87.84 (61.21, 117.76) | −1.04 (−1.25, −0.83) |  | 718.59 (559.81, 890.9) | 0.31 (0.3, 0.33) |  | 44.99 (35.3, 55.94) | 0.39 (0.36, 0.42) |
| Lithuania | 0.92 (0.75, 1.07) | −7.04 (−7.47, −6.6) |  | 34.41 (29.17, 40.56) | −6.99 (−7.37, −6.62) |  | 159.06 (136.45, 187.4) | −3.16 (−3.57, −2.76) |  | 8.69 (7.57, 10.09) | −3.42 (−3.76, −3.07) |
| Luxembourg | 1.1 (0.95, 1.22) | −1.88 (−2.11, −1.66) |  | 21.76 (19.48, 24.09) | −2.31 (−2.46, −2.16) |  | 48.52 (40.32, 58.07) | −0.98 (−1.81, −0.15) |  | 3.9 (3.36, 4.53) | −0.95 (−1.74, −0.15) |
| Madagascar | 7.17 (4.24, 10.26) | −1.33 (−1.39, −1.28) |  | 269.57 (185.51, 368.9) | −1.11 (−1.15, −1.06) |  | 1596.03 (1252.59, 2006.69) | 0.18 (0.17, 0.18) |  | 112.37 (87.31, 142.48) | 0.17 (0.16, 0.17) |
| Malawi | 3.07 (2.19, 4.26) | −1.92 (−2.13, −1.71) |  | 155.07 (114.59, 205.14) | −1.27 (−1.39, −1.16) |  | 1670.5 (1311.23, 2079.91) | 0.16 (0.15, 0.16) |  | 117.56 (90.26, 147.95) | 0.15 (0.14, 0.15) |
| Malaysia | 0.81 (0.64, 0.96) | −3.77 (−4.08, −3.46) |  | 69.91 (54.33, 90) | −2.22 (−2.52, −1.92) |  | 822.53 (654.29, 1013.56) | 0.13 (0.09, 0.16) |  | 47.29 (37.23, 59.69) | 0.02 (−0.02, 0.05) |
| Maldives | 0.48 (0.39, 0.57) | −6.36 (−6.62, −6.09) |  | 55.86 (41.25, 76.62) | −4.05 (−4.33, −3.76) |  | 764.79 (590.92, 948.71) | −0.13 (−0.15, −0.1) |  | 45.3 (35.8, 57.5) | −0.25 (−0.27, −0.23) |
| Mali | 2.51 (1.74, 3.29) | −3.33 (−3.72, −2.93) |  | 131.59 (97.94, 172.91) | −2.43 (−2.75, −2.11) |  | 1194 (926.99, 1483.51) | 0.03 (0.01, 0.05) |  | 84.65 (66.13, 106.77) | −0.01 (−0.03, 0.02) |
| Malta | 0.92 (0.77, 1.06) | −2.44 (−2.53, −2.34) |  | 19.83 (17.35, 22.43) | −2.39 (−2.49, −2.29) |  | 20.45 (16.92, 24.6) | −1.88 (−2.36, −1.39) |  | 2.24 (1.98, 2.54) | −2.06 (−2.34, −1.77) |
| Marshall Islands | 11.08 (7.52, 16.24) | −2.04 (−2.13, −1.94) |  | 481.48 (338.84, 679.42) | −1.68 (−1.79, −1.57) |  | 1027.14 (808.97, 1255.26) | 0.08 (0.07, 0.08) |  | 71.83 (56.4, 88.98) | 0.07 (0.06, 0.07) |
| Mauritania | 1.98 (1.34, 2.62) | −4.33 (−4.68, −3.99) |  | 104.08 (78.02, 136.67) | −2.95 (−3.24, −2.66) |  | 1105.54 (861.58, 1364.74) | 0.04 (0.01, 0.06) |  | 72.97 (56.94, 91.26) | 0.02 (0, 0.04) |
| Mauritius | 0.62 (0.57, 0.66) | −3.57 (−3.98, −3.16) |  | 64.98 (50.14, 84.71) | −1.58 (−1.88, −1.27) |  | 786.67 (620.98, 982.3) | −0.06 (−0.09, −0.03) |  | 46.19 (36.22, 58.34) | −0.13 (−0.15, −0.11) |
| Mexico | 0.63 (0.42, 0.74) | −5.96 (−6.14, −5.78) |  | 38.99 (28.9, 51.42) | −4.39 (−4.58, −4.2) |  | 470.31 (378.5, 576.91) | −0.57 (−0.61, −0.53) |  | 29.82 (23.77, 37.12) | −0.62 (−0.66, −0.58) |
| Micronesia (Federated States of) | 9.59 (6.56, 14.08) | −2.36 (−2.49, −2.22) |  | 410.26 (293.91, 590.35) | −2.1 (−2.22, −1.98) |  | 1031.06 (817.69, 1265.04) | 0.06 (0.05, 0.06) |  | 72.24 (56.98, 90) | 0.06 (0.05, 0.06) |
| Monaco | 0.58 (0.43, 0.77) | −1.38 (−1.58, −1.18) |  | 13.21 (10.14, 17.25) | −1.41 (−1.58, −1.23) |  | 33.21 (26.99, 40.6) | −0.44 (−0.51, −0.36) |  | 2.58 (2.18, 3.13) | −0.58 (−0.66, −0.51) |
| Mongolia | 3.45 (2.86, 4.1) | −3.42 (−3.68, −3.17) |  | 159.11 (128.89, 196.63) | −2.88 (−3.08, −2.68) |  | 1061.51 (819.93, 1331.9) | −0.04 (−0.05, −0.03) |  | 71.32 (55.81, 89.69) | −0.17 (−0.19, −0.15) |
| Montenegro | 1.47 (1.12, 1.87) | −1.48 (−1.73, −1.22) |  | 38.4 (29.89, 47.84) | −1.92 (−2.2, −1.64) |  | 59.89 (49.56, 72.08) | 0.05 (−0.05, 0.16) |  | 4.64 (4, 5.42) | −0.37 (−0.42, −0.31) |
| Morocco | 1.94 (1.42, 2.6) | −2.1 (−2.19, −2.02) |  | 93.23 (70.88, 122.17) | −2.13 (−2.19, −2.07) |  | 751.15 (578.32, 939.46) | 0.02 (0, 0.04) |  | 48.83 (37.86, 62.21) | −0.04 (−0.07, −0.02) |
| Mozambique | 5.63 (4, 7.68) | −0.45 (−0.6, −0.29) |  | 213.78 (161.84, 279.57) | −0.39 (−0.51, −0.27) |  | 1658.2 (1297.07, 2090.53) | 0.04 (−0.02, 0.1) |  | 116.31 (90.4, 148.16) | −0.04 (−0.1, 0.02) |
| Myanmar | 1.87 (1.37, 2.63) | −4.65 (−4.87, −4.42) |  | 125.32 (95.5, 165.57) | −3.9 (−4.07, −3.73) |  | 861.59 (671.43, 1073.34) | −0.12 (−0.17, −0.06) |  | 60.1 (47.14, 75.9) | −0.28 (−0.32, −0.23) |
| Namibia | 2.26 (1.55, 3.14) | −3.08 (−3.25, −2.91) |  | 140.42 (106.71, 185.8) | −2.01 (−2.11, −1.91) |  | 1436.62 (1126.77, 1789.71) | −0.05 (−0.06, −0.04) |  | 91.74 (71.79, 116.5) | −0.1 (−0.11, −0.09) |
| Nauru | 10.35 (7.55, 13.43) | −1.64 (−2.05, −1.23) |  | 435.45 (320.66, 570.39) | −1.45 (−1.89, −1.01) |  | 198.33 (168.61, 232.23) | −0.69 (−0.74, −0.64) |  | 18.16 (16.17, 20.26) | −0.86 (−1.02, −0.69) |
| Nepal | 16.97 (11.54, 26.79) | −2.14 (−2.36, −1.92) |  | 489.93 (343.92, 726.51) | −2.37 (−2.57, −2.17) |  | 692.51 (543.5, 875.13) | 0.18 (0.11, 0.25) |  | 48.43 (37.91, 61.42) | 0.14 (0.07, 0.22) |
| Netherlands | 0.87 (0.74, 0.97) | 0.16 (−0.93, 1.27) |  | 14.88 (13.23, 16.4) | −0.77 (−1.57, 0.04) |  | 30.05 (24.13, 35.86) | −0.11 (−0.31, 0.09) |  | 2.78 (2.39, 3.24) | 0.05 (−0.24, 0.35) |
| New Zealand | 1.65 (1.44, 1.82) | −3.06 (−3.55, −2.57) |  | 44.46 (40.43, 48.37) | −3.23 (−3.64, −2.81) |  | 56.25 (51.06, 61.83) | −0.37 (−0.49, −0.26) |  | 4.92 (4.42, 5.48) | −1.09 (−1.15, −1.04) |
| Nicaragua | 0.48 (0.39, 0.62) | −4.73 (−4.85, −4.62) |  | 73.36 (50.29, 105.72) | −1.83 (−1.93, −1.74) |  | 1233.46 (977.69, 1531.17) | 0.02 (0, 0.05) |  | 76.28 (59.39, 96.58) | −0.05 (−0.07, −0.02) |
| Niger | 3.63 (2.3, 5.61) | −3.16 (−3.46, −2.86) |  | 152.78 (105.3, 218.98) | −2.76 (−3.04, −2.48) |  | 1090.18 (842.39, 1366.57) | 0.13 (0.11, 0.16) |  | 79.02 (60.62, 100.01) | 0.14 (0.12, 0.16) |
| Nigeria | 1.69 (1.31, 2.27) | −4.99 (−5.16, −4.82) |  | 107.58 (78.85, 140.88) | −3.27 (−3.45, −3.09) |  | 1214.14 (944.1, 1516.06) | 0.34 (0.31, 0.38) |  | 80.69 (62.81, 101.77) | 0.27 (0.25, 0.3) |
| Niue | 6.59 (5.45, 8.04) | −2.02 (−2.3, −1.74) |  | 309.67 (258.55, 372.66) | −1.7 (−2.14, −1.27) |  | 148.14 (125.93, 173.23) | −0.72 (−0.76, −0.69) |  | 11.27 (9.97, 12.72) | −1.29 (−1.35, −1.23) |
| North Macedonia | 1.69 (1.21, 2.18) | −3.66 (−3.89, −3.43) |  | 39.8 (29.25, 50.37) | −4.15 (−4.38, −3.91) |  | 55.25 (45.34, 66.57) | −1.42 (−1.56, −1.29) |  | 4.31 (3.75, 5.01) | −2.18 (−2.34, −2.01) |
| Northern Mariana Islands | 3.17 (2.61, 3.69) | −0.57 (−0.76, −0.37) |  | 158.02 (128.37, 193.8) | −0.37 (−0.48, −0.26) |  | 1112.16 (875.8, 1364.99) | 0.15 (0.11, 0.19) |  | 62.07 (49.29, 77.36) | 0.24 (0.2, 0.27) |
| Norway | 0.79 (0.68, 0.87) | −3.59 (−4.85, −2.32) |  | 12.96 (11.62, 14.03) | −3.84 (−5.05, −2.62) |  | 21.94 (18.37, 25.97) | −2.19 (−3.04, −1.34) |  | 2.11 (1.79, 2.47) | −2.89 (−3.69, −2.09) |
| Oman | 1.07 (0.59, 1.44) | −3.31 (−3.58, −3.04) |  | 30.95 (20.33, 39.75) | −3.59 (−3.83, −3.36) |  | 58.14 (47.92, 69.69) | 0.45 (0.32, 0.59) |  | 4.39 (3.72, 5.07) | 0.46 (0.26, 0.65) |
| Pakistan | 17.85 (13.13, 25.81) | −1.67 (−1.92, −1.41) |  | 584.99 (443.3, 816.86) | −1.43 (−1.68, −1.18) |  | 991.01 (789.76, 1235.97) | 0.38 (0.23, 0.52) |  | 68.51 (53.96, 86.55) | 0.36 (0.21, 0.5) |
| Palau | 7.75 (6.2, 9.63) | −1.31 (−1.42, −1.2) |  | 319.25 (253.41, 397.5) | −1.05 (−1.15, −0.95) |  | 158.96 (134.18, 185.39) | −0.66 (−0.71, −0.6) |  | 12.61 (11.14, 14.19) | −1.09 (−1.16, −1.03) |
| Palestine | 1.3 (1.07, 1.53) | −3.09 (−3.17, −3) |  | 71.23 (56.38, 91.15) | −2.24 (−2.34, −2.13) |  | 734.68 (582.31, 903.18) | 0 (−0.02, 0.01) |  | 44.99 (34.95, 56.25) | −0.03 (−0.05, −0.01) |
| Panama | 0.53 (0.41, 0.64) | −5.57 (−5.84, −5.3) |  | 71.81 (50.76, 100.82) | −2.25 (−2.41, −2.08) |  | 1161.41 (916.04, 1431.12) | 0.01 (0, 0.02) |  | 66.4 (51.96, 83.28) | −0.15 (−0.16, −0.13) |
| Papua New Guinea | 13.12 (6.79, 25.41) | −1.64 (−1.69, −1.58) |  | 576.41 (345.5, 1003.24) | −1.43 (−1.5, −1.36) |  | 1006.12 (785.66, 1232.36) | 0.12 (0.09, 0.15) |  | 69.31 (54.23, 85.57) | 0.09 (0.07, 0.12) |
| Paraguay | 0.85 (0.64, 1.1) | −1.84 (−1.95, −1.74) |  | 85.38 (62.83, 117.03) | −0.89 (−0.94, −0.84) |  | 1211.11 (962.75, 1489.21) | 0.02 (0.01, 0.02) |  | 72.25 (55.74, 90.19) | −0.05 (−0.06, −0.04) |
| Peru | 0.58 (0.41, 0.79) | −4.05 (−4.38, −3.71) |  | 70.64 (49.58, 101.14) | −1.48 (−1.65, −1.3) |  | 1130.47 (892.78, 1416.67) | 0.12 (0.09, 0.15) |  | 69.44 (54.01, 88.33) | −0.01 (−0.02, 0.01) |
| Philippines | 1.03 (0.68, 1.51) | −2.16 (−2.29, −2.03) |  | 77.52 (55.74, 104.43) | −1.33 (−1.49, −1.17) |  | 626.91 (486.59, 780.13) | 0.35 (0.26, 0.43) |  | 42.3 (33.16, 52.86) | 0.3 (0.21, 0.39) |
| Poland | 1.39 (1.2, 1.53) | −6.28 (−6.87, −5.69) |  | 32.16 (28.26, 35.31) | −7.13 (−7.69, −6.57) |  | 52.45 (46.03, 59.8) | −3.47 (−4.58, −2.36) |  | 4.68 (4.13, 5.38) | −3.89 (−4.52, −3.25) |
| Portugal | 1.23 (1.06, 1.36) | −3.16 (−3.48, −2.84) |  | 25.93 (23.63, 28.33) | −3.62 (−4.02, −3.22) |  | 35.83 (30.43, 42.29) | −1.74 (−1.95, −1.52) |  | 3.18 (2.84, 3.62) | −2.07 (−2.26, −1.88) |
| Puerto Rico | 0.4 (0.33, 0.48) | −3.26 (−3.71, −2.81) |  | 17.95 (15.18, 21.41) | −3.13 (−3.58, −2.68) |  | 44.87 (37.05, 53.09) | −0.76 (−0.89, −0.62) |  | 2.97 (2.47, 3.47) | −1.14 (−1.32, −0.97) |
| Qatar | 0.89 (0.66, 1.14) | −4.33 (−4.6, −4.05) |  | 24.87 (19.5, 31.43) | −4.13 (−4.29, −3.96) |  | 65.92 (54.83, 78.39) | −1 (−1.07, −0.94) |  | 5.07 (4.38, 5.89) | −0.86 (−0.94, −0.78) |
| Republic of Korea | 0.49 (0.38, 0.59) | −3.6 (−3.76, −3.43) |  | 9.35 (7.88, 10.77) | −4.09 (−4.21, −3.96) |  | 25.59 (20.72, 30.45) | −1.13 (−1.25, −1.02) |  | 1.57 (1.33, 1.82) | −1.49 (−1.61, −1.36) |
| Republic of Moldova | 1.14 (0.97, 1.32) | −6.78 (−7.2, −6.37) |  | 40.41 (34.76, 46.76) | −6.94 (−7.34, −6.54) |  | 121.65 (103.28, 142.93) | −2.22 (−2.29, −2.15) |  | 6.89 (5.95, 8) | −2.86 (−2.96, −2.77) |
| Romania | 1.45 (1.21, 1.68) | −5.56 (−6.09, −5.03) |  | 37.45 (31.16, 43.33) | −6.29 (−6.83, −5.75) |  | 76.73 (63.14, 90.86) | −1.03 (−1.53, −0.53) |  | 5.44 (4.58, 6.42) | −2.09 (−2.36, −1.83) |
| Russian Federation | 1.07 (0.97, 1.16) | −6.55 (−6.92, −6.18) |  | 33.37 (30.03, 37.41) | −7.09 (−7.47, −6.7) |  | 157.93 (137.73, 179.65) | −2.02 (−2.12, −1.91) |  | 9 (7.9, 10.12) | −2.47 (−2.59, −2.35) |
| Rwanda | 2.94 (1.82, 4.15) | −4.24 (−4.64, −3.83) |  | 144 (102.11, 198.54) | −3.17 (−3.49, −2.84) |  | 1568.27 (1234.88, 1965.05) | 0.02 (0.01, 0.03) |  | 109.36 (85.1, 137.4) | −0.03 (−0.04, −0.02) |
| Saint Kitts and Nevis | 0.57 (0.47, 0.7) | −4.22 (−4.55, −3.88) |  | 22.81 (18.81, 27.74) | −4.6 (−4.98, −4.22) |  | 51.61 (42.81, 61.14) | −1.86 (−2.06, −1.67) |  | 3.63 (3.14, 4.23) | −2.1 (−2.31, −1.88) |
| Saint Lucia | 1.25 (1.01, 1.54) | −4.11 (−4.43, −3.78) |  | 105.53 (82.47, 138) | −2.28 (−2.48, −2.08) |  | 1211.51 (955.08, 1499.13) | 0 (−0.02, 0.01) |  | 74 (57.81, 93.96) | −0.05 (−0.06, −0.03) |
| Saint Vincent and the Grenadines | 1.56 (1.34, 1.83) | −2.63 (−2.89, −2.36) |  | 120.31 (96.3, 151.7) | −1.74 (−1.92, −1.56) |  | 1175.98 (918.53, 1449.35) | 0 (−0.01, 0.01) |  | 74.72 (57.64, 94.84) | −0.08 (−0.09, −0.06) |
| Samoa | 6.64 (4.82, 9.56) | −1.2 (−1.4, −1) |  | 298.49 (220.94, 422.66) | −0.84 (−1.01, −0.66) |  | 1090.66 (852.19, 1338.28) | 0.08 (0.06, 0.1) |  | 70.52 (54.83, 88.75) | 0.03 (0.01, 0.05) |
| San Marino | 1.02 (0.66, 1.45) | −2.36 (−2.71, −2) |  | 22.33 (15.21, 30.92) | −2.1 (−2.39, −1.8) |  | 47.17 (38.8, 56.85) | −0.64 (−0.67, −0.61) |  | 4.81 (4.19, 5.46) | −0.77 (−0.83, −0.7) |
| Sao Tome and Principe | 3.88 (2.57, 6) | −2.26 (−2.57, −1.96) |  | 157.43 (110.42, 223.18) | −1.9 (−2.18, −1.63) |  | 1192.46 (938.04, 1475.95) | 0.12 (0.1, 0.15) |  | 76.35 (59.51, 96.71) | 0.08 (0.06, 0.11) |
| Saudi Arabia | 0.73 (0.5, 0.96) | −4.08 (−4.21, −3.95) |  | 26.11 (18.52, 34.34) | −4.1 (−4.23, −3.96) |  | 57.08 (47.34, 67.56) | −0.8 (−0.89, −0.72) |  | 3.89 (3.31, 4.59) | −1 (−1.1, −0.89) |
| Senegal | 2.28 (1.6, 3.18) | −3.6 (−3.96, −3.23) |  | 116.9 (84.16, 156.97) | −2.65 (−2.94, −2.36) |  | 1136.08 (878.03, 1425.25) | −0.04 (−0.07, −0.01) |  | 77.42 (60.18, 97.2) | −0.04 (−0.07, −0.01) |
| Serbia | 1.14 (0.85, 1.41) | −4.02 (−4.33, −3.7) |  | 28.25 (21.61, 34.7) | −3.94 (−4.22, −3.66) |  | 54.68 (44.7, 66.52) | −0.53 (−0.68, −0.38) |  | 4.26 (3.67, 5.05) | −1.32 (−1.49, −1.16) |
| Seychelles | 0.47 (0.39, 0.55) | −2.73 (−2.88, −2.58) |  | 56.28 (41.2, 76.44) | −1.4 (−1.51, −1.29) |  | 778.89 (613.62, 960.16) | −0.13 (−0.15, −0.11) |  | 46.74 (36.88, 58.86) | −0.14 (−0.16, −0.12) |
| Sierra Leone | 2.81 (2, 3.9) | −2.9 (−3.02, −2.78) |  | 137.22 (101.39, 185.49) | −2.1 (−2.24, −1.97) |  | 1132.58 (878.92, 1422.96) | 0.12 (0.11, 0.13) |  | 81.52 (63.15, 103.57) | 0.14 (0.12, 0.16) |
| Singapore | 0.56 (0.47, 0.63) | −4.52 (−4.63, −4.41) |  | 11.5 (10.28, 12.67) | −4.99 (−5.06, −4.91) |  | 25.19 (20.87, 29.81) | −3.25 (−3.56, −2.94) |  | 1.38 (1.17, 1.62) | −3.84 (−4.2, −3.49) |
| Slovakia | 1.06 (0.87, 1.28) | −3.49 (−3.69, −3.29) |  | 31 (25.71, 37.09) | −3.65 (−3.8, −3.5) |  | 96.33 (80.09, 115.98) | −0.5 (−1.19, 0.2) |  | 6.56 (5.62, 7.84) | −0.6 (−1.08, −0.11) |
| Slovenia | 2.16 (1.77, 2.51) | −3.71 (−4.19, −3.23) |  | 39.79 (33.5, 45.99) | −5.26 (−5.77, −4.75) |  | 58.41 (47.87, 71.62) | −1.85 (−2.51, −1.18) |  | 5.82 (5.12, 6.73) | −1.66 (−2.02, −1.3) |
| Solomon Islands | 10.02 (5.4, 17.03) | −1.66 (−1.73, −1.6) |  | 403.11 (244.29, 646.18) | −1.38 (−1.45, −1.3) |  | 1002.32 (775.78, 1239.08) | 0.07 (0.06, 0.08) |  | 74.08 (57.71, 91.81) | 0.07 (0.06, 0.08) |
| Somalia | 5.83 (3.49, 10.99) | −1.86 (−2.01, −1.71) |  | 224.34 (153.39, 357.23) | −1.53 (−1.65, −1.4) |  | 1615.82 (1261.09, 2023.59) | 0.16 (0.15, 0.17) |  | 121.45 (94.72, 153.12) | 0.19 (0.18, 0.19) |
| South Africa | 1.62 (1.45, 1.85) | −2.43 (−2.87, −2) |  | 127.1 (98.4, 167.57) | −1.68 (−1.96, −1.41) |  | 1541.14 (1217.47, 1923.9) | 0 (−0.01, 0.01) |  | 100.94 (78.24, 127.7) | −0.03 (−0.05, −0.02) |
| South Sudan | 4.47 (3.19, 6.08) | −1.51 (−1.79, −1.23) |  | 194.81 (149.65, 257.8) | −1.01 (−1.26, −0.76) |  | 1593.53 (1248.25, 2013.94) | 0.24 (0.22, 0.26) |  | 110.85 (85.8, 140.64) | 0.3 (0.26, 0.34) |
| Spain | 1.92 (1.58, 2.17) | −3.18 (−3.3, −3.06) |  | 36.82 (32.08, 40.64) | −3.78 (−3.94, −3.63) |  | 55.48 (46.64, 66.25) | −1.12 (−1.3, −0.94) |  | 5.32 (4.72, 5.99) | −1.59 (−1.68, −1.5) |
| Sri Lanka | 0.53 (0.35, 0.75) | −4.19 (−4.33, −4.04) |  | 24.98 (17.94, 33.63) | −4.12 (−4.3, −3.95) |  | 62.94 (51.79, 75.72) | −0.51 (−0.62, −0.39) |  | 4.57 (3.88, 5.32) | −1.05 (−1.17, −0.93) |
| Sudan | 2.57 (1.58, 4.15) | −3.21 (−3.29, −3.13) |  | 120.91 (82.77, 179.3) | −3.41 (−3.49, −3.32) |  | 628.31 (489.07, 769.29) | 0.14 (0.12, 0.15) |  | 44.19 (34.41, 55.02) | 0.05 (0.03, 0.06) |
| Suriname | 0.95 (0.75, 1.19) | −2.72 (−2.95, −2.49) |  | 94.47 (72.09, 124.13) | −1.53 (−1.7, −1.36) |  | 1119.2 (872, 1382.83) | −0.02 (−0.03, −0.01) |  | 71.3 (54.99, 90.47) | −0.09 (−0.11, −0.07) |
| Sweden | 0.64 (0.54, 0.72) | −3.11 (−3.42, −2.79) |  | 10.6 (9.35, 11.85) | −3.46 (−3.77, −3.14) |  | 18.14 (15.29, 21.33) | −3.04 (−3.66, −2.42) |  | 1.91 (1.64, 2.21) | −2.79 (−3.26, −2.32) |
| Switzerland | 0.75 (0.64, 0.83) | −3.51 (−3.68, −3.33) |  | 15.87 (14.45, 17.4) | −3.46 (−3.58, −3.34) |  | 39.91 (32.81, 48.63) | −1.99 (−2.22, −1.75) |  | 3.06 (2.6, 3.63) | −2.73 (−2.88, −2.57) |
| Syrian Arab Republic | 1.94 (1.35, 2.69) | −3.99 (−4.41, −3.56) |  | 91.75 (67.6, 121.31) | −3.62 (−4.02, −3.23) |  | 718.27 (564.75, 887.83) | 0.01 (0, 0.03) |  | 46.14 (36.24, 57.07) | −0.01 (−0.04, 0.01) |
| Taiwan (Province of China) | 0.6 (0.51, 0.67) | −6.86 (−7.44, −6.27) |  | 19.03 (16.02, 22.3) | −5.8 (−6.33, −5.27) |  | 140.35 (120.32, 162.26) | −1.22 (−1.46, −0.98) |  | 8.43 (7.27, 9.63) | −1.75 (−2.03, −1.47) |
| Tajikistan | 1.85 (1.38, 2.48) | −2.57 (−2.84, −2.29) |  | 116.61 (88.66, 154.84) | −1.81 (−1.96, −1.66) |  | 1061.51 (832.73, 1343.73) | 0.01 (−0.01, 0.03) |  | 70.51 (54.48, 90.12) | 0.03 (−0.02, 0.09) |
| Thailand | 0.36 (0.29, 0.46) | −3.09 (−3.39, −2.79) |  | 50.6 (37.22, 70.15) | −1.5 (−1.66, −1.35) |  | 723.7 (568.89, 892.54) | −0.08 (−0.09, −0.07) |  | 45.64 (35.68, 57.83) | −0.17 (−0.19, −0.15) |
| Timor−Leste | 2.35 (1.27, 4.17) | −2.53 (−2.88, −2.17) |  | 139.34 (87.6, 232.56) | −2.12 (−2.47, −1.76) |  | 787.38 (614.53, 993.16) | −0.03 (−0.04, −0.02) |  | 52.83 (40.89, 66.5) | −0.13 (−0.16, −0.1) |
| Togo | 2.64 (1.86, 3.76) | −3.19 (−3.37, −3.01) |  | 125.71 (94.31, 167.54) | −2.35 (−2.51, −2.19) |  | 1096.84 (868.9, 1364.79) | 0.02 (0.01, 0.04) |  | 77.95 (61.17, 97.3) | 0.04 (0.02, 0.06) |
| Tokelau | 7.52 (6.06, 9.61) | −3 (−3.29, −2.7) |  | 355.42 (291.67, 454.78) | −2.65 (−3.13, −2.18) |  | 149.44 (126.25, 174.98) | −0.96 (−1.02, −0.9) |  | 12.67 (11.17, 14.43) | −1.66 (−1.74, −1.58) |
| Tonga | 2.86 (2.13, 3.76) | −1.79 (−1.85, −1.72) |  | 171.09 (133.08, 221.41) | −1.16 (−1.23, −1.09) |  | 1503.07 (1183.66, 1844.85) | 0.02 (−0.15, 0.19) |  | 89.29 (69.18, 110.61) | −0.02 (−0.18, 0.15) |
| Trinidad and Tobago | 1.03 (0.75, 1.37) | −3.1 (−3.38, −2.81) |  | 99.87 (76.04, 131.78) | −1.61 (−1.77, −1.46) |  | 1182.66 (931.55, 1468.07) | 0.11 (0.09, 0.12) |  | 70.63 (54.81, 88.61) | −0.07 (−0.09, −0.05) |
| Tunisia | 1.3 (0.88, 2.03) | −1.71 (−1.91, −1.52) |  | 37.42 (24.71, 57.34) | −2.35 (−2.55, −2.14) |  | 53 (44.23, 62.92) | −0.61 (−0.66, −0.57) |  | 3.92 (3.36, 4.58) | −0.73 (−0.76, −0.69) |
| Türkiye | 0.88 (0.72, 1.05) | −2.46 (−3.02, −1.89) |  | 25.36 (21.1, 30.2) | −3.05 (−3.48, −2.62) |  | 49.51 (40.69, 58.54) | −0.53 (−0.69, −0.38) |  | 3.22 (2.69, 3.82) | −0.66 (−0.83, −0.5) |
| Turkmenistan | 2.93 (2.18, 3.9) | −3.59 (−3.96, −3.22) |  | 157.14 (125.33, 196.97) | −2.92 (−3.19, −2.65) |  | 1023.27 (793, 1285.78) | −0.04 (−0.05, −0.03) |  | 65.76 (51.29, 83.61) | −0.16 (−0.19, −0.14) |
| Tuvalu | 8.61 (6.24, 12.02) | −2.9 (−3.03, −2.78) |  | 331.58 (243.95, 458.41) | −2.93 (−3.07, −2.79) |  | 171.55 (143.43, 199.57) | −1.01 (−1.08, −0.93) |  | 17.34 (15.32, 19.53) | −1.55 (−1.63, −1.47) |
| Uganda | 2.38 (1.63, 3.21) | −3.12 (−3.34, −2.89) |  | 130.71 (95.12, 174.49) | −1.86 (−2.03, −1.69) |  | 1554.82 (1210.44, 1922.62) | 0.03 (−0.08, 0.13) |  | 105.8 (82.49, 132.42) | −0.03 (−0.14, 0.08) |
| Ukraine | 0.98 (0.71, 1.27) | −3.5 (−4.28, −2.71) |  | 36.55 (27.77, 46.56) | −3.33 (−4.11, −2.55) |  | 124.26 (106.77, 142.72) | −0.73 (−0.79, −0.67) |  | 7.08 (6.18, 8.04) | −0.98 (−1.06, −0.9) |
| United Arab Emirates | 1.87 (1.14, 2.62) | −1.34 (−1.86, −0.82) |  | 81.31 (60.51, 106.03) | −1.78 (−2.01, −1.55) |  | 699.35 (543.21, 875.71) | −0.04 (−0.08, 0) |  | 40.17 (31.23, 50.69) | 0.08 (0.04, 0.12) |
| United Kingdom | 0.76 (0.67, 0.81) | −4.7 (−4.93, −4.47) |  | 16.06 (14.8, 17.3) | −4.76 (−5, −4.51) |  | 43.56 (36.3, 53.24) | −0.35 (−0.39, −0.31) |  | 4.93 (3.95, 6.03) | −0.35 (−0.39, −0.31) |
| United Republic of Tanzania | 3.23 (2.25, 4.32) | −1.93 (−1.99, −1.88) |  | 154.76 (112.45, 201.75) | −1.14 (−1.18, −1.1) |  | 1668.4 (1307.98, 2086.54) | 0.26 (0.24, 0.28) |  | 110.52 (85.54, 139.7) | 0.17 (0.16, 0.19) |
| United States of America | 0.83 (0.68, 0.91) | −3.38 (−3.94, −2.82) |  | 22.56 (19.64, 25.08) | −3.4 (−3.99, −2.81) |  | 123.44 (112.81, 135.61) | −0.94 (−1.33, −0.54) |  | 10.04 (9.06, 11.04) | −0.83 (−1.29, −0.37) |
| United States Virgin Islands | 0.56 (0.42, 0.72) | −3.01 (−3.19, −2.82) |  | 27.71 (21.63, 35) | −2.55 (−2.74, −2.36) |  | 51.55 (42.33, 61.37) | −0.56 (−0.63, −0.5) |  | 3.44 (2.92, 4.06) | −0.62 (−0.68, −0.56) |
| Uruguay | 0.63 (0.54, 0.7) | −3.44 (−3.6, −3.28) |  | 16.1 (14.25, 17.99) | −3.36 (−3.48, −3.23) |  | 63.8 (52.38, 76.41) | −0.25 (−0.38, −0.11) |  | 4.95 (4.29, 5.73) | −0.64 (−0.78, −0.49) |
| Uzbekistan | 4.82 (3.93, 5.74) | 0.11 (−0.12, 0.34) |  | 206.67 (168.72, 246.57) | −0.37 (−0.56, −0.19) |  | 1060.64 (821.53, 1328.71) | 0.25 (0.22, 0.28) |  | 69.64 (53.99, 87.93) | 0.14 (0.12, 0.16) |
| Vanuatu | 12.52 (8.13, 19.91) | −1.86 (−1.94, −1.78) |  | 532.6 (367.82, 819.53) | −1.57 (−1.66, −1.48) |  | 1132.7 (887.28, 1385.67) | 0.05 (0.04, 0.05) |  | 78.36 (61.12, 97.23) | 0.04 (0.03, 0.05) |
| Venezuela (Bolivarian Republic of) | 0.44 (0.33, 0.58) | −5.63 (−6.03, −5.22) |  | 16.09 (12.28, 20.82) | −5.43 (−5.83, −5.02) |  | 44.87 (37.03, 53.09) | −1.13 (−1.33, −0.94) |  | 3.01 (2.53, 3.49) | −1.39 (−1.61, −1.18) |
| Viet Nam | 0.86 (0.61, 1.18) | −3.34 (−3.48, −3.2) |  | 37.37 (27.74, 48.89) | −3 (−3.23, −2.78) |  | 124.33 (105.99, 145.37) | 1.47 (1.05, 1.9) |  | 8.6 (7.5, 9.77) | 0.72 (0.35, 1.09) |
| Yemen | 3.17 (2.08, 4.97) | −2.84 (−3.01, −2.68) |  | 138.68 (99.52, 194.77) | −2.72 (−2.84, −2.59) |  | 850.27 (668.06, 1062.81) | 0.12 (−0.1, 0.33) |  | 60.23 (46.87, 76.04) | 0.09 (−0.1, 0.29) |
| Zambia | 3.42 (1.87, 5.17) | −2.31 (−2.52, −2.11) |  | 156.24 (106.83, 217.94) | −1.68 (−1.8, −1.56) |  | 1642.06 (1291.83, 2041.87) | 0.02 (−0.01, 0.05) |  | 112.49 (87.59, 142.08) | −0.05 (−0.07, −0.03) |
| Zimbabwe | 7.34 (4.97, 10.02) | 1.29 (0.7, 1.87) |  | 338.98 (246.7, 443.34) | 1.47 (0.95, 1.99) |  | 1417.73 (1112.13, 1754.91) | 0.09 (0.06, 0.12) |  | 97.82 (76.16, 125.11) | 0.18 (0.14, 0.21) |
